# A new ancient lineage of ablepharine skinks (Sauria: Scincidae) from eastern Himalayas with notes on origin and systematics of the group

**DOI:** 10.7717/peerj.12800

**Published:** 2022-01-18

**Authors:** Zeeshan A. Mirza, Andrey M. Bragin, Harshal Bhosale, Gaurang G. Gowande, Harshil Patel, Nikolay A. Poyarkov

**Affiliations:** 1 National Centre for Biological Sciences, Bangalore, Karnataka, India; 2 Department of Vertebrate Zoology, Biological Faculty, Lomonosov Moscow State University, Moscow, Russia; 3 Bombay Natural History Society, Mumbai, Maharashtra, India; 4 Abasaheb Garware College, Pune, Maharashtra, India; 5 Department of Biotechnology, Fergusson College, Pune, Maharasshtra, India; 6 Department of Biosciences, Veer Narmad South Gujarat University, Surat, Gujarat, India; 7 Voluntary Nature Conservancy, Vallabh Vidyanagar, Gujarat, India; 8 Joint Russian-Vietnamese Tropical Research and Technological Center, Hanoi, Nghia Do, Cau Giay, Vietnam

**Keywords:** *Ablepharus*, *Asymblepharus*, Herpetology, *Himalblepharus*, MicroCT, Molecular phylogeny, Reptiles, Systematics

## Abstract

The Himalayas represent a renowned biodiversity hotspot and an important biogeographic realm that has influenced origin and diversification of multiple taxa. A recent herpetological investigation of the eastern Himalayas of the Indian state of Arunachal Pradesh led to the discovery of a unique lineage of ablepharine skink, which is herein described as a new genus along with a new species. The findings are based an integrated taxonomic approach incorporating data from external morphology, microCT scans of the skull and molecular data. The molecular phylogeny of ablepharine skinks is also presented that suggests taxonomic amendments. Discovery of this unique lineage of skinks further highlights the biogeographic importance of the eastern Himalayas as a source for origin of several relic biota.

## Introduction

The family Scincidae is a diverse group of mostly diurnal lizards (Smith, 1935) with around 1,727 species distributed in various habitats across the eastern and western hemisphere (Uetz, Freed & Hošek, 2021). In India, the family is represented by more than 60 species across 17 genera and data on even the most common and widespread species is lacking, especially concerning their generic allocation (Deuti et al., 2020; Uetz, Freed & Hošek, 2021). Many of the currently known genera have overlapping characters and at times, assigning a species to a particular genus becomes difficult without molecular data. Furthermore, molecular data for most species, especially type species of respective genera are unavailable, making generic allocation of most species problematic.

In the course of a herpetological expedition, we visited Talle Valley Wildlife Sanctuary, Arunachal Pradesh State, India (see Bhosale, Gowande & Mirza, 2019; Mirza et al., 2020) where we collected several specimens of an unknown species of a scincid lizard. From its external morphology, the species superficially showed affinity to the members of the genera *Asymblepharus* Eremchenko & Szczerbak, 1980 and *Scincella* Mittleman, 1950. We generated molecular data for four mitochondrial gene fragments for the collected specimens and representatives of the genera *Asymblepharus* (Eremchenko & Szczerbak, 1980), *Ablepharus* Lichtenstein, 1823 and *Scincella*. To assess the identity and phylogenetic placements of the newly collected specimens from the Talle Valley, we also generate molecular data for a few representatives of *Asymblepharus*, *Ablepharus* and *Scincella*, including respective type species of the genera *Asymblepharus* and *Ablepharus* in order to assess phylogenetic relationships among the group. Comparison of the details of external morphology, including scalation, skull features and molecular data, suggested that the species represents a deeply divergent and undescribed lineage of ablepharine skinks warranting recognition as a separate genus; this outcome was further supported by molecular data; our findings further shed new light on the systematics of the group. Herein, we describe the newly collected specimens as a new species and further propose a new genus to embody the species and its closest relatives from the Eastern Himalaya while providing notes on the systematics of the ablepharine skinks.

## Materials and Methods

### Nomenclatural acts

The electronic version of this article in Portable Document Format (PDF) will represent a published work according to the International Commission on Zoological Nomenclature (ICZN), and hence the new names contained in the electronic version are effectively published under that Code from the electronic edition alone (see Articles 8.58.6 of the Code). This published work and the nomenclatural acts it contains have been registered in ZooBank, the online registration system for the ICZN. The ZooBank LSIDs (Life Science Identifiers) can be resolved and the associated information can be viewed through any standard web browser by appending the LSID to the prefix http://zoobank.org/. The LSID for this publication is as follows: urn:lsid:zoobank.org:pub:BE943DD6-3FBD-4A1B-B967-DCC2D9DAA175. The online version of this work is archived and available from the following digital repositories: PeerJ, PubMed Central and CLOCKSS.

### Fieldwork and specimen acquisition

Specimen collection protocols and animal operations followed the Institutional Ethical Committee of Wildlife Protection and Research Society (certificate number 243/2019 of 25/05/2019).

Field sampling, including collection of samples and animals in the field, was authorized by the Department of Environment and Forest, Government of Arunachal Pradesh (permits CWL/Gen/173/2018-19/Pt.V11/2421-33 and CWL/Gen/173/2018-19/Pt.V11/2434-43).

Specimens of the new species were collected on field with hand, photographed and later, euthanized with halothane within 24 h of capture, following ethical guidelines for animal euthanasia (Leary et al., 2013). The specimens were fixed in 8% formaldehyde solution and later stored in 70% ethanol. Liver/tail tip tissues were collected for molecular work and stored in molecular grade ethanol prior to specimen fixation. The specimens have been deposited in the collection of the Bombay Natural History Society (BNHS), Mumbai and the Research Collection Facility of the National Centre for Biological Sciences (NCBS), Bangalore (Mirza et al., 2020, 2021).

### Morphological data

Sex of the collected specimens was determined by everting the hemipenis. The following morphometric data were collected using a Mitutoyo digital caliper with an error of 0.1 mm: SVL (snout to vent length, measured from the tip of the snout to the cloacal opening), Ax-Gr length (distance between the axilla to the groin), BW (Body width measured at the midpoint at the widest portion of the trunk), CL (measured from base of heel to knee with the knee bent), TL (tail length), TW (tail width measured at tail base), SnL (Snout length measured from snout tip to the angle of the jaw), HL (head length measured from the snout tip to the anterior border of the tympanic opening), HW (Head width measured at the widest portion at the angle of the jaw), HH (head height measured at the highest point of the head at the angle of the jaw), FL (measured from the base of palm to elbow when it is bent), OD (eye diameter measured at its widest), NE (distance from nares to the anterior border of the eye), EE (distance from the posterior border of the eye to the anterior border of the tympanic opening), EL (ear diameter measured at its widest), IN (inter narial/nose distance measured between the nostrils), IO (inter-orbital/eyes distance), SnFl (measured from the snout tip to the forelimb anterior insertion).

MicroCT scans were generated for the male specimen BNHS 2853 using a Bruker® Skyscan 1272 (Bruker BioSpin Corporation, Billerica, MA, USA). Head of the specimen was scanned for 210 min at resolution of 3 µm and recording data for every 0.4° rotation for 360° with (AL) 1 mm filter. The source voltage for the scan was 65 kV and source current was 153 uA. Three-dimensional reconstructions were generated using CTVox (Bruker BioSpin Corporation, Billerica, MA, USA) and Amira 6.7 (Thermo Fisher Scientific, Waltham, MA, USA); skull bones, as well as an endocast of the inner ear, were digitally segmented, and images were further edited in Adobe Photoshop CS6. Osteological description is based on volume renders retrieved from CTVox following terminology of the skull described by Evans (2008). The microCT scan is uploaded to MorphoSource accessible at the following link: https://www.morphosource.org/concern/biological_specimens/000381778.

### Molecular analysis

Genomic DNA was isolated from the preserved tissues of the type specimens using QIAGEN DNeasy kits following protocols directed by the manufacturer. A partial fragment of the mitochondrial 16S rRNA (*16S*), 12S rRNA (*12S*), cytochrome b (*cyt*b), NADH dehydrogenase subunit 2 (*ND*2) and three nuclear melanocortin 1 receptor (*MC1R*) and NK-tumor recognition (*NKTR*) and recombination activating gene 1 (RAG1) genes/mitochondrial non-coding regions were amplified using primers listed in Table 1. A 22.4 µl reaction was set for a bi-directional Polymerase Chain Reaction (PCR), containing 10 µl of Thermo Scientific DreamTaq PCR Master Mix, 10 µl of molecular grade water, 0.2 µl of each 10 µM primer and 2 µl template DNA, carried out with an Applied Biosystems ProFlex PCR System. Thermo-cycle profile used for amplification were as follows: 95 °C for 3 min, (denaturation temperature 95 °C for 30 s, annealing time ranged from 40 to 50 s, elongation temperature 72 °C for 1 min) × 36 cycles, 72 °C for 10 min, hold at 4 °C. PCR product was cleaned using QIAquick PCR Purification Kit and sequenced with an Applied Biosystems 3730 DNA Analyzer. Available sequences of *RAG1* gene for Scincidae were downloaded and a preliminary ML phylogeny analysis was run to ascertain the phylogenetic position of the new genus following methods described below and strategize sampling for a more robust phylogenetic analysis (Fig. S1). Based on the results recovered from *RAG1* phylogeny, additional sequences were included in the analysis. In addition to this, *ND2*, *cytb*, *16S rRNA*, *12S rRNA*, *MCR1*, and *NKTR* genes sequences of scincid lizards available on GenBank® were downloaded for molecular phylogenetic reconstructions (Table S1). Sequences were aligned in MegaX (Kumar et al., 2018) using ClustalW (Thompson, Higgins & Gibson, 1994) with default settings. The sequences were concatenated using SequenceMatrix (Vaidya, Lohman & Meier, 2011). Taxa for molecular phylogenetics were selected based on the preliminary *RAG1* tree and the tree topologies recovered by Pyron, Burbrink & Wiens (2013). *Ateuchosaurus chinensis Gray, 1845* was chosen as an outgroup for the analysis based on the phylogenetic results of Pyron, Burbrink & Wiens (2013). For optimal partitioning strategy and evolutionary substitution model, the aligned data was analyzed using PartitionFinder v. 1.1.1 (Lanfear et al., 2012), implementing a greedy search algorithm under the Akaike Information Criterion (AIC). Molecular phylogenetic analysis follows methods described by Mirza et al. (2020, 2021). The aligned dataset was subject to phylogenetic inference using IQ-TREE (http://iqtree.cibiv.univie.ac.at/) online portal (Minh et al., 2020). Sequence substitution model was selected using the auto parameter with provision for FreeRate heterogeneity and the analysis was run with an ultrafast bootstrap option for 1,000 iterations to assess clade support (Table S2). Bayesian Inference (BI) was implemented in MrBayes 3.2.2. (Ronquist & Huelsenbeck, 2003) and was run for 10 million generations and sampled every 1,000 generations. BI run included five parallel chains, three hot and tow cold chains. The standard deviation of split frequencies of the analysis reached were below 0.01, after which the analysis was not continued further. Twenty-five percent of trees generated were discarded as burn-in. Data were subjected to Bayesian Inference phylogenetic reconstructions with model of the sequence substitution model, based on the optimal partitioning scheme suggested by PartitionFinder (Table S2). Trees were further visualized using FigTree v.1.4.3. (Rambaut, 2012).

**Table 1:** Details of primers used in the study for PCR amplification and sequencing.

Gene	Primer name	Primer sequence (5′-3′)	Amplicon size	Annealing temperature	Reference
*16s rRNA*	16Sa 16Sb	CGCCTGTTTATCAAAAACAT CCGGTCTGAACTCAGATCACGT	523	45	Palumbi et al., 1991
12S rRNA	12Sa 12Sb	AAACTGGGATTAGATACCCCACTAT GAGGGTGACGGGCGGTGTGT	~380	48	Kocher et al., 1989
*cytb*	L14910 H16064	CCATCCAACATCTCAGCATGATGAAA CCCTCAGAATGATATTTGTCCTCA	310	48	Kocher et al., 1989
*ND*2	LJXND1-ND2R LJXND1-ND2F H4980 L4437	AGTAGYAGGYGRCAGGTTGT CTMACCYTATTTTTCGTAGT TGGGTTTGRTTAAGCTTTCGGGC CCATACCAAGCTTTCGGGC	400–800	54	Xu et al., 2020; Macey et al., 1997
*RAG1*	R13 R18	TCTGAATGGAAATTCAAGCTGTT GATGCTGCCTCGGTCGGCCACCTTT	1,023	58	Groth & Barrowclough, 1999
*MCR1*	MC1RF MC1RR	AGGCNGCCATYGTCAAGAACCGGAACC CTCCGRAAGGCRTAAATGATGGGGTCCAC	668	56	Pinho et al., 2010
*NKTR*	NKTRf19 NKTRr18	GATGACATGGAGATYTGYACTCC CTYCTDGAYCGACTTCTTGAGTGACT	580	50	Townsend et al., 2011

## Results

### Molecular analysis

**Figure 1:**
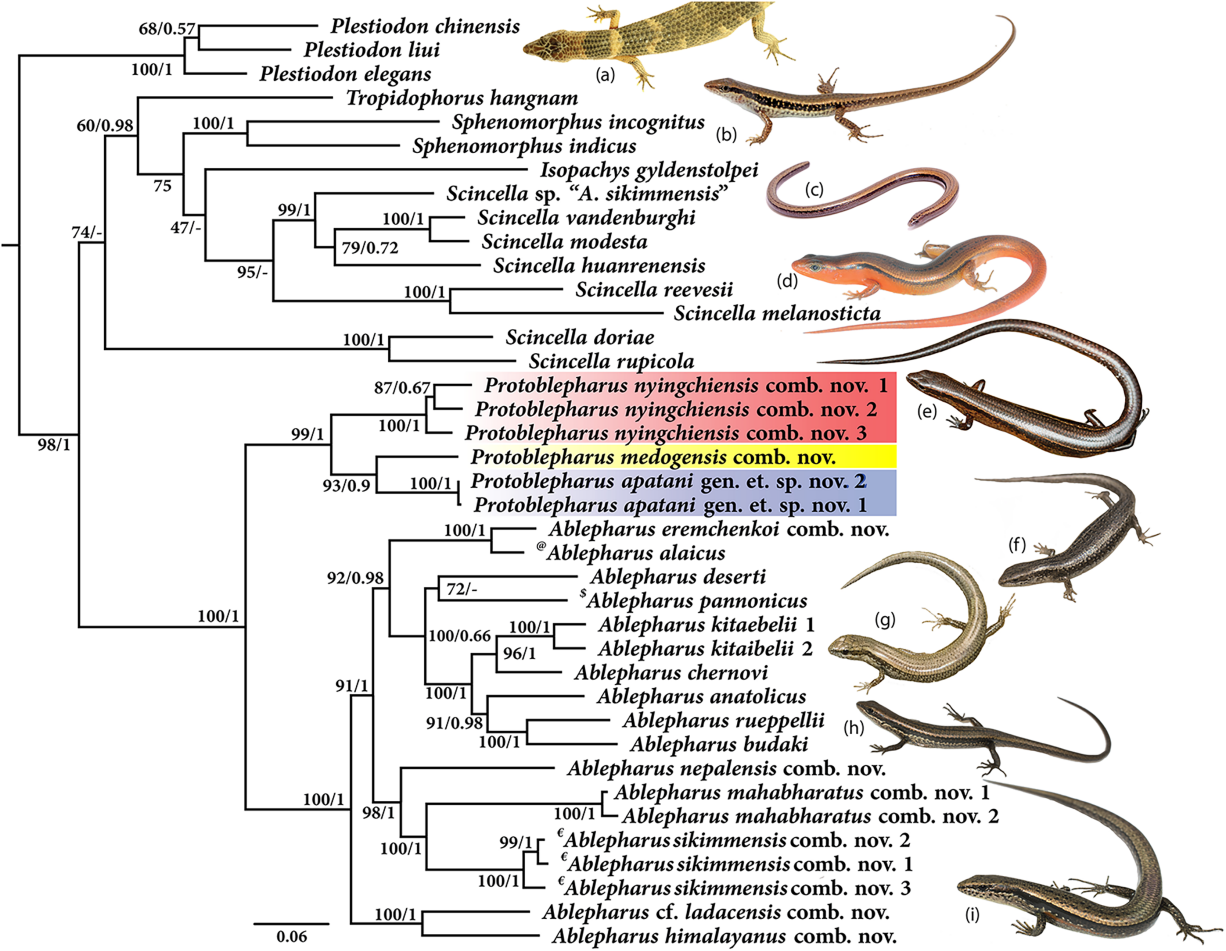
Maximum likelihood phylogeny tree for selected scincid genera based on concatenated 4,244 bp of mitochondrial (12S, 16S, *cyt* b, *ND*2) and nuclear (MC1R & NKTR) genes. The new genus and its members are highlighted in colour: ‘red’ *Protoblepharus nyingchiensis* comb. nov., ‘yellow’ *Protoblepharus medogensis* comb. nov., and ‘blue’ *Protoblepharus apatani* gen. et. sp. nov. Numbers at nodes indicate ML-based bootstrap support followed by the Bayesian posterior probability values. Species denoted by symbol indicate the type species of: ‘@’ *Asymblepharus*, ‘$’ *Ablepharus*, ‘€’ subgenus *Himalblepharus*. Photos (A) *Tropidophorus* sp. by Zeeshan A. Mirza, (B) *Sphenomorphus indicus* by Nikolay A. Poyarkov, (C) *Isopachys gyldenstolpei* by Nikolay A. Poyarkov, (D) *Scincella rufocaudata* by Nikolay A. Poyarkov, (E) *Protoblepharus apatani* gen. et. sp. nov. by Zeeshan A. Mirza, (F) *Ablepharus alaicus* by Andrey M. Bragin, (G) *Ablepharus pannonicus* by Andrey M. Bragin, (H) *Ablepharus nepalensis* comb nov. by Andrey M. Bragin, (I) *Ablepharus sikkimensis* comb. nov. by Andrey M. Bragin.

Molecular phylogeny was based on four mitochondrial *12S rRNA, 16S rRNA, cytb*, *ND2* and two nuclear *MC1R* and *NKTR* genes for 40 species (Fig. 1). The concatenated dataset comprised of 4,244 bp of which mitochondrial dataset consisted of 2,958 bp whereas the nuclear dataset comprised of 1,286 bp. The analysis recovered *Plestiodon Duméril & Bibron, 1839* clade as a sister to a strongly supported group (98/1.0, hereafter support values are given for ML bootstrap/BI posterior probabilities, respectively) including two sister clades. One of the latter contained *Tropidophorus Duméril & Bibron, 1839*, *Sphenomorphus Fitzinger, 1843*, *Isopachys Lönnberg, 1916*, and *Scincella*. The second clade further consisted of two subclades (100/1), one containing the newly collected specimens from Arunachal Pradesh along with two *Asymblepharus* species from Medog Region of Tibet, China (*As. medogensis* + *As. nyingchiensis*). The remaining species of *Ablepharus* + *Asymblepharus* and members of the genus *Ablepharus* belonged to the second subclade (100/1.0, see Fig. 1). The specimens of ablepharine skinks from the eastern Himalaya (*As. medogensis*, *As. nyingchiensis*, and the Arunachal Pradesh population) formed a strongly supported group (99/1.0) which forms a sister clade to all remaining species of *Ablepharus* + *Asymblepharus* (100/1.0). The Arunachal Pradesh population formed a strongly supported sister lineage to *As. medogensis*. The type species of the genus *Asymblepharus*, *As. alaicus* (Elpatjevsky, 1901) and sampled congeners as well as the type species of the subgenus *Himalblepharus* Eremchenko, 1987, *i.e., Asymblepharus* (*Himalblepharus*) *sikimmensis* (Blyth, 1854) were found to be embedded within *Ablepharus*, rendering the latter paraphyletic.

### Systematics

Originally the ablepharine skinks of Mediterranean and Central Asia with the complete fusion of eyelids were assigned to the genus *Ablepharus* Fitzinger in Lichtenstein (1823), while the Himalayan taxa with free eyelids were assigned to the genus *Scincella *Mittleman, 1950 (Eremchenko & Szczerbak, 1986). Eremchenko & Szczerbak (1980) reviewed taxonomy of ablepharine lizards in the former USSR and established a separate genus *Asymblepharus* Eremchenko & Szczerbak, 1980 for *A. alaicus* Elpatjevsky, 1901, a species in which the eyelids are only partially fused. Later, Eremchenko (1987) has expanded the genus *Asymblepharus*, to which it was included along with *As. alaicus* the five species of Himalayan *Scincella* with free eyelids, and proposed to recognize a separate subgenus *Himalblepharus* for the Himalayan taxa (type species–*As. sikkimensis*), while *As. alaicus* was retained in the nominative subgenus *Asymblepharus*. Though this taxonomy got widely accepted (Uetz, Freed & Hošek, 2021), the limits and phylogenetic relationships among *Ablepharus*, *Asymblepharus*, *Himalblepharus* and *Scincella* remain vague.

In the present study we provide the most complete phylogeny for the ablepharine skinks, which includes representatives of all major currently recognized lineages (see Fig. 1). Our phylogeny indicates the presence of a deep split between the ablepharine skinks from the eastern Himalaya (*As. medogensis*, *As. nyingchiensis* and the newly discovered population from Arunachal Pradesh) and all the remaining ablepharine skinks, which form two well-supported reciprocally monophyletic groups (Fig. 1). Within the latter subclade, the species of ablepharine skinks from the Mediterranean and Middle Asia form a clade (92/0.98) deeply nested within the radiation of Himalayan taxa (100/1). The type species of the subgenus *Himalblepharus* (*As. sikkimensis*) is grouped with *As. mahabharatus* and *As. nepalensis* (98/1.0), while *As. himalayanus* and *As*. cf. *ladacensis* form a strongly supported clade (100/1) with sister relationships to all remaining species of *Ablepharus* and *Asymblepharus* (Fig. 1). At the same time, *As. alaicus*, the type species of the subgenus *Asymblepharus*, is grouped with its sister species *As. eremchenkoi*, and together these taxa form a sister lineage to the clade including the species of *Ablepharus sensu* stricto, though with high support (92/0.98) (Fig. 1).

Our findings advocate merging of the two presently recognized genera of ablepharine skinks and we herein treat both *Asymblepharus* Eremchenko & Szczerbak 1980 **syn. nov.** and *Himalblepharus* Eremchenko, 1987 **syn. nov.** as subjective junior synonyms of *Ablepharus* Fitzinger, 1824 in accordance to the Article 23 of the code (International Commission on Zoological Nomenclature, 1999). The recently described species *Asymblepharus medogensis* Jiang, Wu, Guo, Li & Che, 2020 and *Asymblepharus nyingchiensis* Jiang, Wu, Wang, Ding & Che, 2020 from Medog, Tibet, were found to form a well-supported clade (99/1.0) together with the newly discovered population from Talle Valley (Arunachal Pradesh), which has sister relationships with *Ablepharus sensu lato*. Below we argue that the molecular and morphological differentiation of the ablepharine clade from the Eastern Himalaya, including the Arunachal Pradesh and Medog populations warrants elevation to a genus level, and thereby we describe it as a new genus below. Within the members of this clade, both species recently described from Medog (*A. medogensis* and *A. nyingchiensis*) differ from the species from Talle Valley Wildlife Sanctuary genetically as well as morphologically. The systematic status of the population from Arunachal Pradesh as well as the other closely related ablepharine skinks is discussed in detail in the subsequent sections.

***Protoblepharus* gen. nov.**

[urn:lsid:zoobank.org:act:2FF9E503-7E77-42E3-85EA-521CD3F260BC]

**Types species:** *Protoblepharus apatani* **sp. nov.** [described below].

**Species included:** *Protoblepharus apatani* **sp. nov.**; *Protoblepharus medogensis* **comb. nov.** Jiang, Wu, Guo, Li and Che, 2020; *Protoblepharus nyingchiensis* **comb. nov.** Jiang, Wu, Wang, Ding and Che, 2020.

**Phylogenetic definition:** *Protoblepharus* is a maximum crown-clade name referring to the clade originating with the most recent common ancestor of *Protoblepharus apatani* **sp. nov.** and *Protoblepharus nyingchiensis* **comb. nov.**, and includes all extant species that share a more recent common ancestor with these taxa than with *Ablepharus pannonicus*.

**Etymology:** The proposed generic epithet is a Latinized combination of the Greek words ‘*proto*’ (*πρωτό*), meaning ‘primitive’ or ‘first’, and ‘*blepharo*’ (*βλέφαρο*) meaning ‘eyelid’ referring to the ablepharine skinks. The proposed name highlights the distinct phylogenetic position of the new genus with reference to *Ablepharus* skinks.

**Recommended English name:** East-Himalayan Skinks.

**Diagnosis:** Ablepharine skinks with a small to medium sized body (SVL up to 63 mm) with a subtle posterior palatine process. The pterygoid lacking process at the palatine-pterygoid junction and furthermore, pterygoid bordering the suborbital fossa. Postorbital bone present. The pterygoid lacking a recurved posterior process. Supranasal bone absent. Lower eyelid scaly and movable, lacking a transparent “window”. Body scales glossy, smooth or striated, lacking keels in 23–26 rows around the midbody. Scales on dorsum and body flanks with or without apical pits. Tympanum with or without small lobules. No reduction of digits (pentadactyle). Paravertebral scales 53–64. Lamellae on fourth finger 7–10 and 11–15 (rarely 16) on fourth toe. A single large scale covering the dorsal surfaces of each digit. Medial pair of precloacal scales enlarged.

For a detailed description of the cranial morphology see the account on the *Protoblepharus apatani* **sp. nov.** described below.

**Comparisons:** The new genus is compared to members of the clade that include the following genera: *Ablepharus*, *Scincella*, *Isopachys*, *Sphenomorphus* and *Tropidophorus* (see Fig. 1). The new genus, *Protoblepharus* **gen. nov.** differs from the genus *Ablepharus* and *Scincella* in bearing a subtle posterior palatine process (*vs* posterior palatine process absent in *Ablepharus* and *Scincella*), lower eyelid movable (*vs* fused in some species of *Ablepharus*) lacking the transparent “window” (*vs* present in some species of *Ablepharus* and *Scincella*). The new genus further differs from *Tropidophorus* in bearing a sunken tympanum (*vs* tympanum exposed and superficial in *Tropidophorus*), in dorsal scales smooth and feebly striated and glossy (*vs* heavily keeled in *Tropidophorus*). The new genus further differs from *Isopachys* in having well-developed limbs (*vs* absent in *Isopachys*). The new genus differs from the members of the genus *Sphenomorphus* having pterygoid bones widely separated, not in contact (*vs* in contact in *Sphenomorphus*), it further differs from some members of the genus *Sphenomorphus* in lacking supranasal bone (*vs* present in a few species of *Sphenomorphus*).

**Figure 2:**
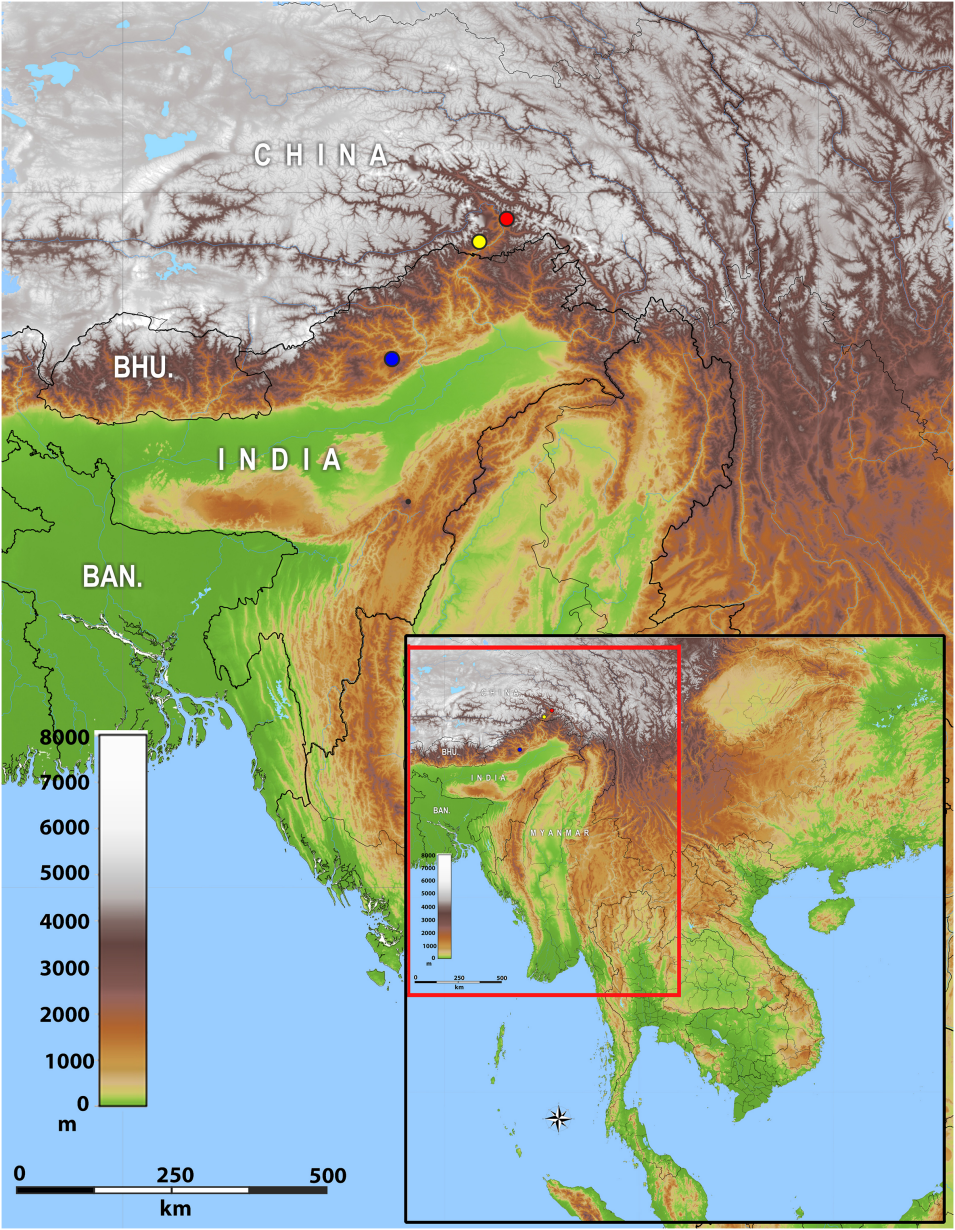
Map of showing the distribution of *Protoblepharus* gen. nov. in Arunachal Pradesh, India and Tibet, China. Coloured circles indicate the type localities: blue solid circle Talle Valley Wildlife Sanctuary, India *(Protoblepharus apatani* **gen. et sp. nov.**); yellow solid circle Medog, Tibet, China (*Protoblepharus medogensis* **comb. nov.**); red solid circle Medog, Tibet, China (*Protoblepharus* *nyingchiensis* **comb. nov.**).

**Distribution:** The new genus inhabits montane forests of Eastern Himalaya from Arunachal Pradesh State of India eastwards along the Brahmaputra River valley to Medog County of Tibet (Xizang) in China (Fig. 2).

***Protoblepharus apatani* gen. et. sp. nov.**

[urn:lsid:zoobank.org:act:A0776D1F-1399-47EC-8A10-96CE76E56290]

Figures 1–7; Figure S1; Table 2

**Figure 3:**
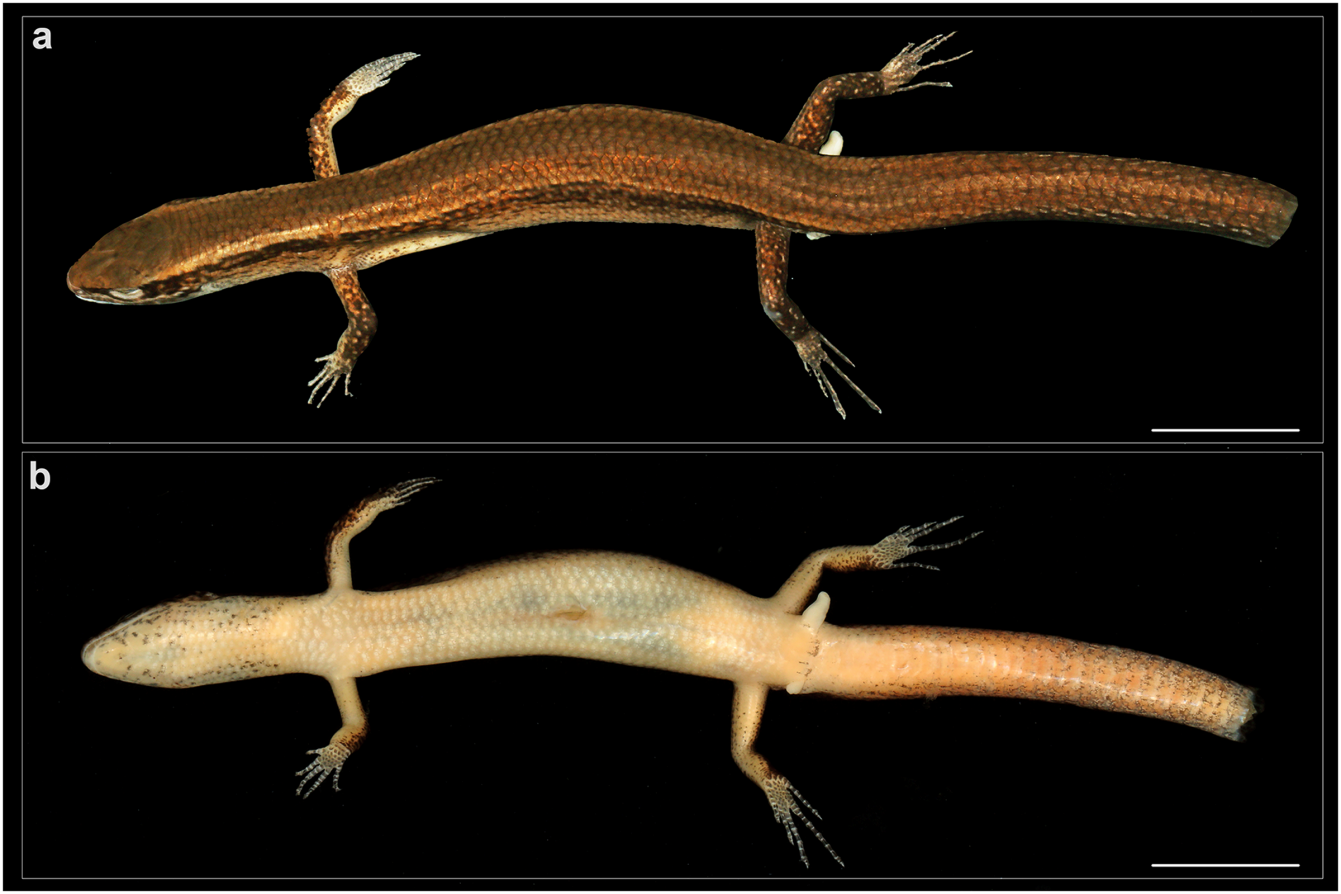
General view of *Protoblepharus apatani* gen. et sp. nov. holotype male (BNHS 2853) in preservative. (A) Dorsal view, (B) ventral view. Scale bar 10 mm. Photos by: Zeeshan A. Mirza.

**Figure 4:**
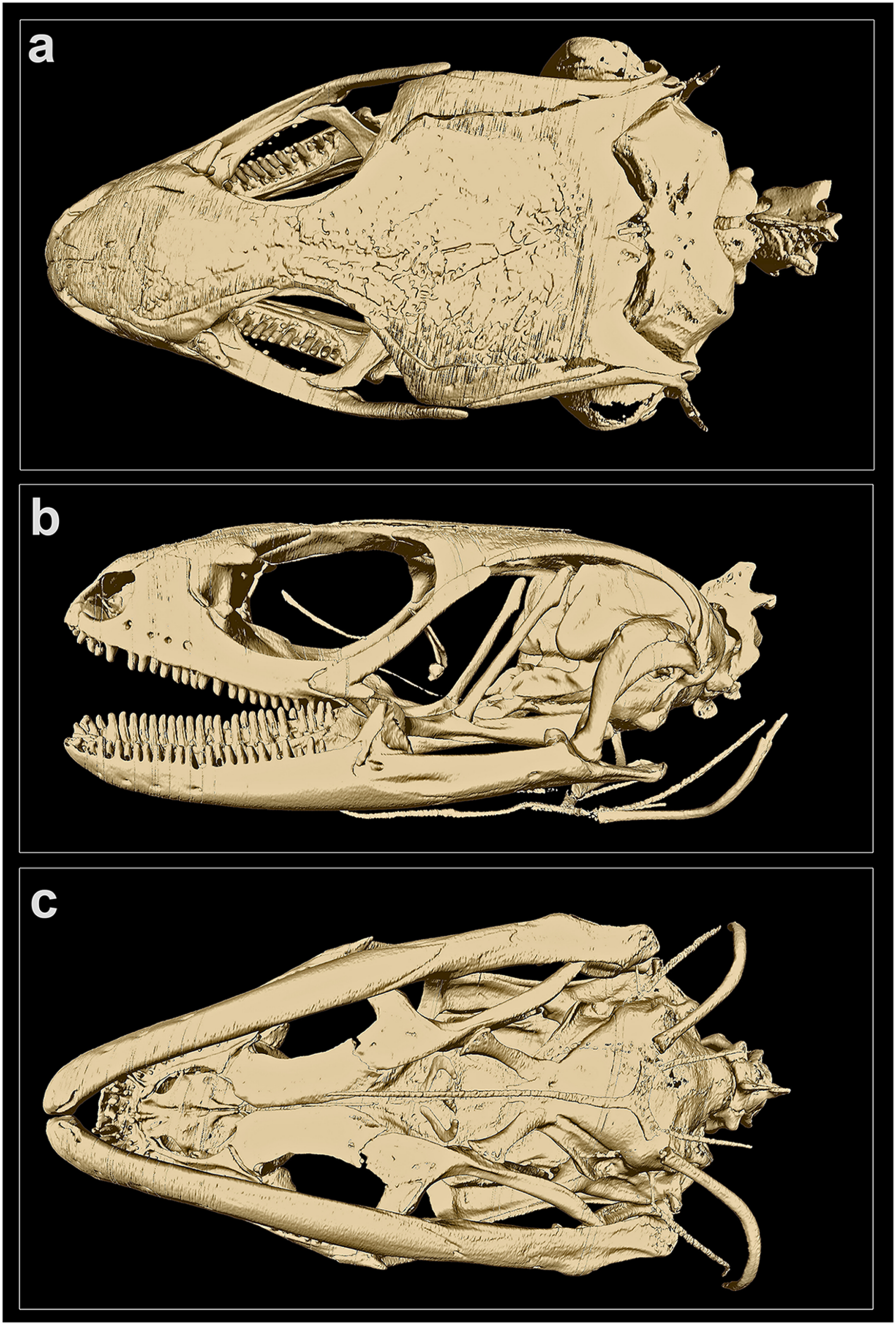
MicroCT-scan of the skull of the holotype of *Protoblepharus apatani* gen. et sp. nov. (BNHS 2853) with the osteoderm cover removed (visualized with CTVox and Amira 6.7). (A) Cranium, dorsal view; (B) cranium, left lateral view; (C) cranium, ventral view.

**Figure 5:**
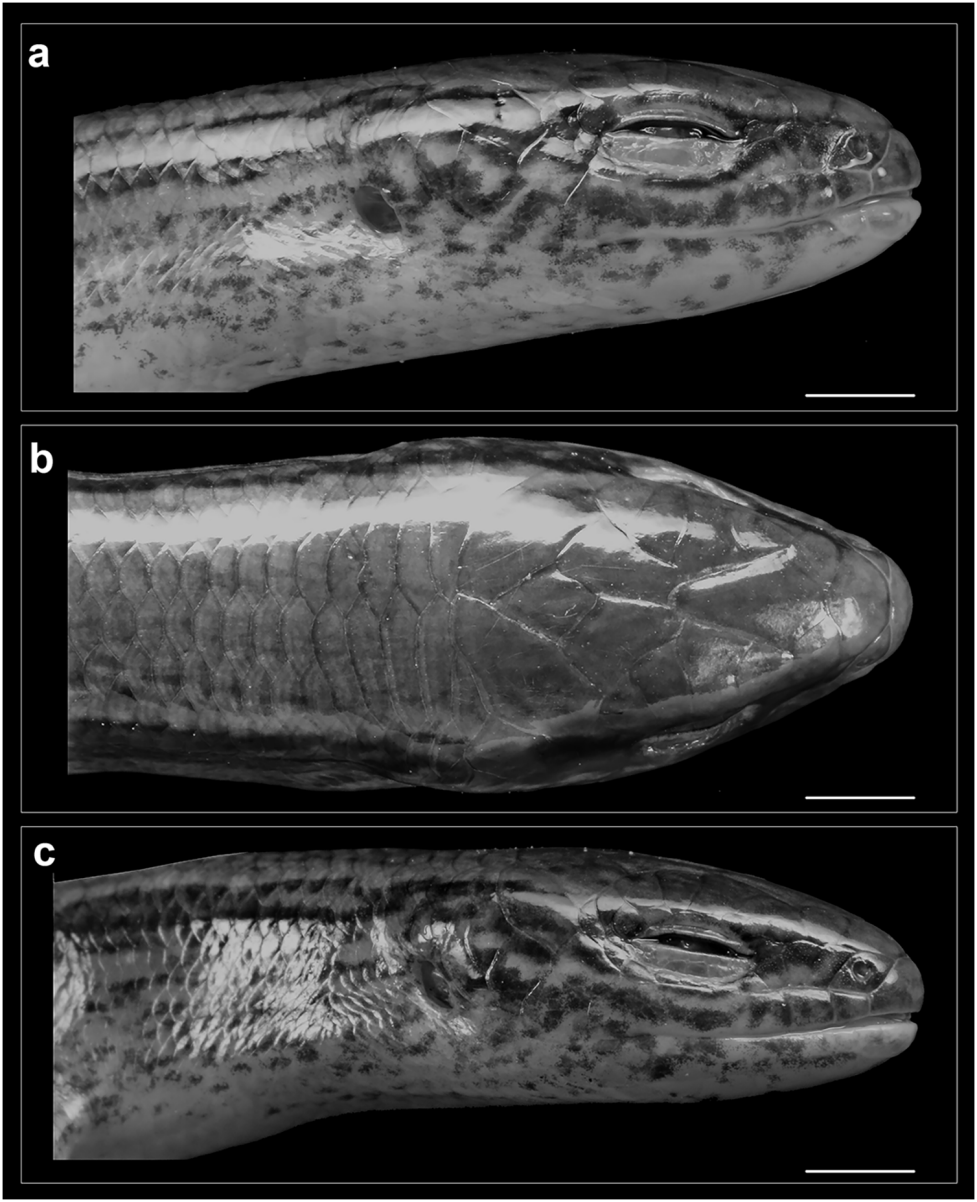
Head of *Protoblepharus apatani* gen. et sp. nov. holotype male (BNHS 2853) in preservative. (A) Head right lateral view, (B) dorsal view, (C) left lateral view. Scale bar 2 mm. Photos by: Zeeshan A. Mirza.

**Figure 6:**
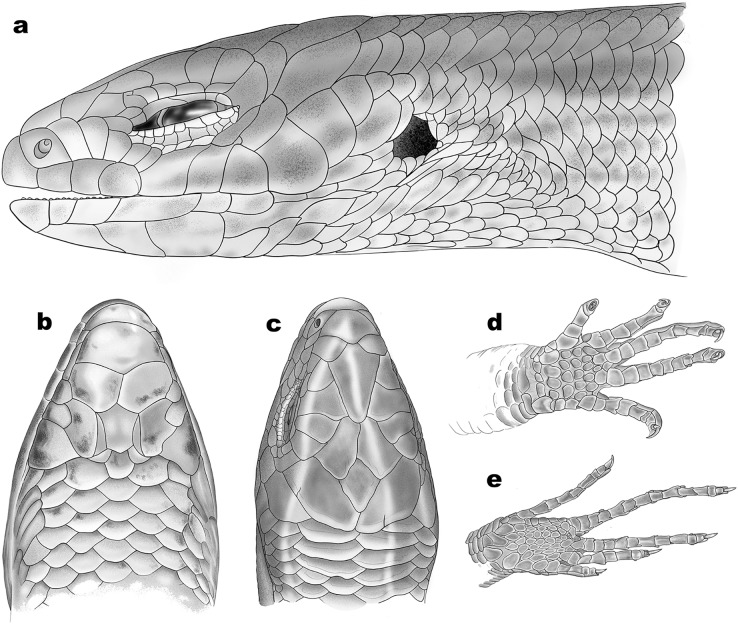
Schematic representation of scalation of *Protoblepharus apatani* gen. et sp. nov. (A) Head lateral view, (B) ventral view, (C) dorsal view, (D) ventral view of manus, (E) ventral view of pes. Drawings by: Hrishikesh Hardikar.

**Figure 7:**
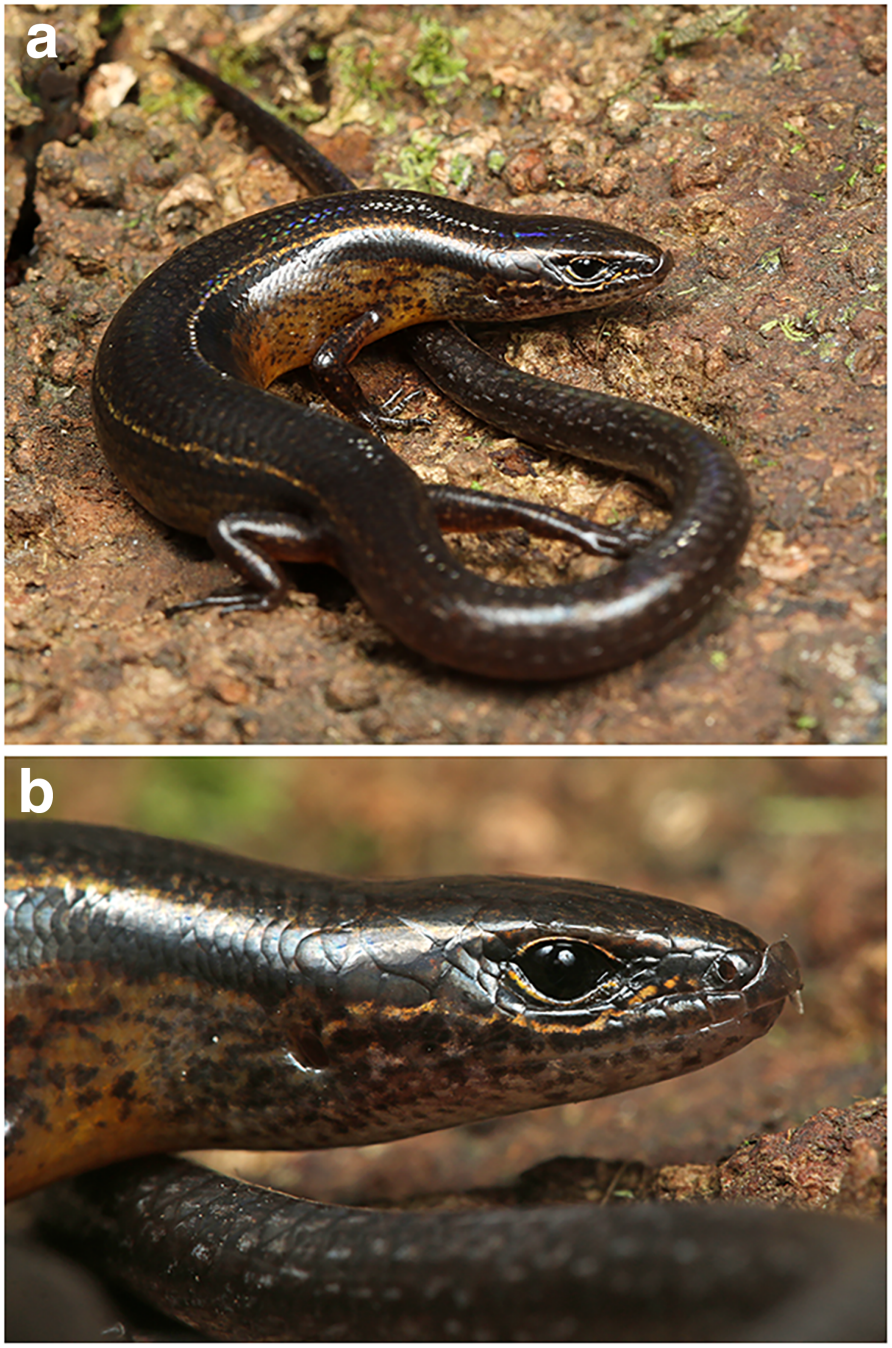
*Protoblepharus apatani* gen. et sp. nov. holotype male (BNHS 2853) in life. Photos by: Zeeshan A. Mirza.

**Table 2:** Measurements and scale counts of *Protoblepharus apatani* gen. et sp. nov. type series. Abbreviations are listed in the Materials and methods.

	Holotype	Paratypes			
Specimen number	BNHS 2853	BNHS 2854	BNHS 2855	NRC-AA-0017	NRC-AA-0018
Sex	Male	male	male	male	female
SVL (snout to vent length)	50.8	43.5	48.8	55.1	40.2
Ax-Gr length	25.3	23.5	26.2	28.4	19.6
BW (Body width)	5.8	6.6	6.3	7	5.8
CL (from base of heel to knee)	5.9	3.5	4.2	5.9	4.3
TL (tail length)	31.2*	70.3	56.6	17*	34*
TW (tail width)	5	3.9	4.7	4.9	3.4
Snout length	5.9	6.3	5.9	7	4.6
HL (head length)	9	7.9	8.5	9.1	7.6
HW (Head width)	5.5	5.5	5.7	6.7	4.9
HH (head height)	4.1	4.4	4.7	5.1	3.5
FL (base of palm to elbow)	4.2	3.6	4.3	4.6	3.4
OD (eye diameter)	2.3	2.6	2.8	2.4	2.3
NE (nose to eye)	2	1.4	1.8	1.9	1.5
SE (snout to eye)	3.3	2.5	2.8	3	2.7
EE (eye to ear)	3.5	3	3.8	3.6	2.6
EL (ear diameter)	1	0.9	1.2	1.1	0.8
IN (inter narial (nose) distance)	1.9	1.9	2.2	2.1	1.7
IO (inter-orbital/eyes)	2.8	2.7	2.9	2.9	2.6
Snout to forelimb	17	15.1	17.3	18.3	15
Lamellae Left manus	5-7-9-10-7	6-8-9-7-5	5-8-9-9-5	5-7-6*-10-7	5-8-9-9-5
Lamellae Left pes	5-8-10-12-8	5-8-9-12-7	5-8-10-12-7	5-9-11-13-5*	5-8-10-12-7
Supralabials Left/Right	6/6	6/6	6/6	6/6	6/6
Infralabials Left/Right	5/5	5/5	5/5	6/5	6/6
Dorsal scale round the mid-body	26	26	26	26	26
Paravertebral scales	56	59	61	57	59
Ventral scales	61	60	57	56	58

**Note:**

The asterisk (*) indicates that the tail was damaged.

**Holotype.** BNHS 2853, adult male, collected from Pange camp Talle Valley Wildlife Sanctuary, 0.64 nautical miles (1.19 km) north of Ziro town, East Kameng district, Arunachal Pradesh (27.547172°N, 93.897038°E, elevation 1,864 m a.s.l, datum WGS84), India by Harshal Bhosale, Pushkar Phansalkar, Mandar Sawant, Gaurang Gowande and Zeeshan Mirza on 10 July 2019.

**Paratypes (*n* = 4).** BNHS 2854–2855, NCBS NRC-AA-0017 three adult males, and NCBS NRC-AA-0018, one adult female with the same collection data as for the holotype.

**Diagnosis:** A small-sized member of the genus *Protoblepharus* **gen. nov.** with adult body size measuring SVL = 40.0–55.1 mm. Dorsal scales striated with apical pits, in 26 rows around the midbody; apical pits present on scales on dorsal surfaces of forelimbs and hindlimbs. Three to four pairs of nuchals. Tympanum lacking lobules. Six supralabial scales; 12–13 subdigital lamellae on fourth toe.

**Comparisons:** The new species, *Protoblepharus apatani* **gen. et. sp. nov.**, differs from *Protoblepharus medogensis* **comb. nov.** in bearing nuchal scales (*vs* absent), lamellae on IV toe 12 (rarely 13) (*vs* 11 lamellae on IV toe); it further differs from *Protoblepharus nyingchiensis* **comb. nov.** in lacking projecting lobules on the anterior border of the ear opening (*vs* 1–7 projecting lobules), lamellae on IV toe 12 (rarely 13) (*vs* 15–16 lamellae on IV toe).

**Etymology:** The specific epithet is given as a noun in apposition honouring the Apatani tribe of Ziro Valley in Arunachal Pradesh; we would like to express our gratitude to the members of the local Apatani community for their help and assistance during our fieldwork in Arunachal Pradesh.

**Recommended English name:** Apatani East-Himalayan Skink.

**Description of holotype** (BNHS 2853, Figs. 3–7): Holotype, an adult male in a good state of preservation, lacking the posterior part of the tail, which was broken during the capture; with a small ca. 3 mm long mid-ventral incision. The left hemipenis of the specimen is partly everted to determine the sex of the specimen. The specimen is preserved in a linear manner with a slight curve to its trunk; the right manus directed posteriorly. (Fig. 3).

SVL 50.8 mm; snout acute in dorsal and lateral aspects (IN/IO ratio 0.68), slightly projecting beyond lower jaws; nostril circular, with lateral orientation, situated much closer to the snout tip than to the eye (NE/SE ratio 0.60); head longer than wide (HL/HW 1.6), slightly tapered in lateral aspect (Fig. 4). Body slender (BW/SVL ratio 0.11); head not distinct from neck, trunk elongated (TRL/SVL ratio 0.50). Forelimbs and hindlimbs relatively short and stout, the tips of digits of forelimb and hindlimb do not meet when the limbs are adpressed against each other along the body axis (Fig. 3); forearm very short (FL/SVL ratio 0.08); tibia longer than FL (CL/SVL ratio 0.12); digits moderately long, slender and ending with distinctly visible, sharp, thin, slightly curved, and pointed claws (Fig. 3). Subdigital lamellae smooth on manus and pes, entire; number of subdigital lamellae including claw sheath: left manus 4-7-9-10-7; left pes 5-8-9-13-8. Relative lengths of digits (measurements in mm in parentheses): left manus IV (2.6) < III (2.5) < V (1.8) < II (1.6) < I (0.8); left pes IV (5.4) < III (4) < II (2.9) < V (2.7) < I (1.5). Scales on dorsal and ventral surfaces of the limbs imbricate with a single row of apical pits.

Rostral wider (3.2 mm) than high (2.6 mm), in contact with first supralabials, nasals and supranasal (Figs. 5, 6). A pair of supranasals not in contact with each other; remotely in contact with slightly smaller postnasals laterally (Fig. 6). A large single frontonasal in broad contact with the rostral anteriorly and the frontal posteriorly; widely in contact with nasal, the anterior loreal, and prefrontal laterally. A pair of prefrontals separated by the frontal, anteriorly in contact with frontonasal, posteriorly in contact with first and second supraoculars, laterally in contact with two loreals. Frontal large, nearly twice longer than wide, irregularly pentagonal in shape with slightly rounded tapering posterior margin, contacting frontonasal and prefrontals anteriorly, in wide contact with second and third supraoculars laterally, in wide contact with frontoparietals posteriorly. Frontoparietals widely in contact with parietals and interparietal scale posterolaterally, contacting frontal and third, fourth and fifth supraoculars anterolaterally. Interparietal rhomboid, posteriorly in contact with parietals; parietal eye indistinct. Parietals anteriorly in contact with frontoparietals, interparietal, fifth supraoculars and laterally touching upper anterior and posterior temporals. Five supraoculars, third being the largest, decreasing in size in both directions (Figs. 5, 6). Scales on the lateral aspect of the head bear pits, throughout their surface and densely place. The density of the pits on the scales is sparse on dorsal head scales and are mostly concentrated towards the posterior borders of each scale (Fig. 5).

Nostril circular, located in the center of nasal. Postnasals small, almost half in size than the anterior loreal, bordered by supranasal, anterior loreal, supralabial I. Anterior and posterior loreals roughly quadrangular, posterior loreal larger than anterior loreal, loreals bordered by postnasal, frontonasal, prefrontal, 1^st^ supraocular and supralabial II. Two preoculars, the upper one smaller than the lower one, two presuboculars, both slightly larger than the upper preoculars (Fig. 5). Upper eyelid bordered by five supraciliaries; and the lower eyelid with 10 ciliary scales. Six supralabials, gradually increasing in size, with the supralabial IV being the largest; supralabials IV and V forming the lower border of the eyelid. Two moderately enlarged scales behind the eye form the postoculars. Two anterior and two posterior temporals, subequal in size, all smooth and cycloid (Figs. 5, 6). Five infralabials, of them the third being the largest. Mental scale wider than long, its posterior margin concave. Anterior postmental single, large, nearly three times the length of mental, anterior postmental widely in contact with infralabials I and II on both sides, a pair of posterior postmentals medially contacting each other, laterally in contact with infralabials II and III. The first pair of adjoining chin shields touching infralabials III–V. The second pair of chin shields separated by a subequal cycloid scale.

Dorsal scalation heterogeneous, composed of smaller cycloid, imbricate scales laterally and a series of slightly enlarged, broad vertebral scales; the latter include three or four pairs of nuchals starting from behind parietals followed by six rows of scales, each nearly half size of a nuchal scale ending at the level slightly beyond the groin; roughly two to four times larger than the adjoining dorsolateral scales. The paravertebral scale series composed of 56 scales including nuchals, with one row of apical pits. Lateral body scales with one row of apical pits. Scales around the midbody in 26 rows. Dorsal scales striated, glossy. Scales on lateral aspects of neck and limbs much smaller and more or less similar in shape to those on body flanks. Ventral scales similar in size and shape to those on the flanks; the midventral scale series composed of 64 scales when counted from chin shield to cloaca. Scales across belly in 8–10 transverse rows. Medial pair of precloacal scales enlarged. Tail broken; tail scales cycloid, smooth, imbricate; all equal in shape except subcaudals, which are greatly widened and by far the largest (twice the size of vertebral row). Scales near the base of the tail in 20–22 rows including the subcaudal scale. Scales on the palmar and plantar regions much smaller than the associated digit lamellae and the scales on ventral surfaces of limbs; rounded or slightly elongated, smooth, and sub-imbricate.

**Colour in preservative:** Golden brown overall with a broad dark stripe running posteriorly from the loreal region all through the lateral surfaces of the head gradually diffusing with the background colour at the mid-body (Fig. 3). The dark lateral stripe is dorsally edged by a light thin stripe running along the upper border of the supralabials till the mid-body. The supralabials with irregular dark blotches (Fig. 5). Ventrally cream-white with a few sparse black spots near the infralabials. The limbs bearing dark reticulate patterns.

**Colour in life:** Overall dorsal coloration in a shade of blackish-brown with a pair of thin golden longitudinal stripes, running from the supraocular to the beginning of the tail (Fig. 7A). A broad black lateral stripe running along the body from the nasals to the tail. A thin golden streak running from the nasals through the supralabials to the opening of the tympanum (Fig. 7B). The dark coloration of the dorsum gradually changing to yellowish-orange towards the ventrolateral edge of the belly eventually turning to yellowish-orange on the venter. The tail dark brown with sparse white speckles (Fig. 7A). All scales are iridescent in life.

**Skull morphology:** In CT-scanned specimen (BNHS 2853, the holotype), osteoderms formed a complete layer, covering almost all skull except the rostral, orbiatal and tympanal regions, therefore disabling the direct observation of the cranial skeleton (Figure S2). The osteoderm cover was nearly completely removed from the skull by digital means (see Fig. 4); however the dorsal surface of the skull-roofing bones looks irregular, with some degree of sculpturing visible on the posterior half of the nasals, on the frontals and on the parietal (Fig. 4A). The interpretation of bone margins on the skull roof is complicated due to the artifacts of the CT-scan visualization, because of the incomplete removal of osteoderms. However, a detailed examination of the CT-scans indicated that the nasals and the frontals are separate, whereas the parietals are fused in BNHS 2853.

The skull is slender, high, comparatively elongated, with a length to width ratio of 1.75, with its greatest width across the quadrates (Fig. 4A). The premaxillae are separated along the midline by a vertical suture, continuing posteriorly by the sutures between the internasals and interfrontals. The narial openings are sub-ovoid, dorso-posteriorly delimitated by the anterior margins of the nasals; anteriorly and medially delimitated by the lateral portion of the nasal process and by the maxillary process of the premaxilla, respectively; and laterally bordered by the anterior margin of the maxillae (Fig. 4B). The narial floor medially formed by the maxillary processes of the premaxilla, posteriorly by the septomaxilla, and laterally by the anterior processes of the maxilla. The nasals subpentagonal, posteriorly contacting the frontals, forming posterolateral processes contacting the maxillae and the prefrontals laterally. The supranasal bones absent.

The frontals are much wider than the nasals anteriorly, gradually narrowing posteriorly reaching the narrowest part at the level of the middle of the orbit, and widening again further posteriorly where they contact the anterior edge of the parietal (Fig. 4A). Anteriorly the frontal contacting the posterior process of the prefrontal, posteriorly it contacts the anteromedial process of the postfrontal. The frontals contribute to the middle one third of the orbital margin edging it with their lateral margins (Fig. 4B). The orbit is anteriorly edged by the lacrimal, anteroventrally by the posterior portion of the maxilla, dorsally by the prefrontal, frontal and postfrontal bones. The anterior margin of the orbit is interrupted at the point where the maxilla and the prefrontal meet by the palpebral bone forming a triangular expansion with postero-lateral orientation (Figs. 4A, 4B). Postero-ventrally the orbit is edged by the posterodorsal process of the jugal, connecting the posterior process of the maxilla to the postorbital (Fig. 4B). A small section of the postorbital anterior process contacts the tip of the posterodorsal process of the jugal (Fig. 4B).

The parietal is as long as wide with a length to width ratio of 1.01. Parietal anteriorly of the same width as the combined posterior margins of the frontals, parietal lateral edges continuing posteriorly almost parallel to the body axis and then they widen slightly posteriorly to the temporal fenestrae. Each temporal fenestra is an longitudinally elongated slit, medially edged by the lateral margin of the parietal, anteriorly by the posterior margin of the postfrontal, anterolaterally by the postorbital, posterolaterally by the squamosal. The neurocranium is convex in dorsal view and is only partially covered by the parietal; the posterior part of the neurocranium is formed by fused supraoccipital and exoccipitals; a small medial incision formed by the opening of the foramen magnum (Fig. 4A); ventrally defined by the basioccipital forming the condylus occipitalis (Fig. 4B).

The bones of the palatal complex are visible in ventral view (Fig. 4C). The palatal complex displays the following openings: the vomeronasal openings, the suborbital fossae, and the interpterygoid vacuity (ordered anteroposteriorly). The vomers are separate forming a medial longitudinal suture between them. Vomeronasal openings small, paired, teardrop-shaped, and are defined by the anterolateral borders of the vomers medially, and by the anteromedial margins of the palatal shelfs of the maxillae laterally (Fig. 4C). The palatines are wide, completely separated along their midline, each forming a subtle poorly ossificated posterior palatine process, each broadly contacting the pterygoids (Fig. 4C). The ectopterygoids sickle-shaped, medially connecting to the petrygoids; laterally connecting to the posterior part of the maxillae. The pterygoids are slightly longer than the vomers and the palatines together; lacking processes at the palatine-pterygoid junction. The suborbital fossae are paired, elongated almond-shaped openings; each fossa is defined posteriorly and postero-medially by the anterior expansion of the pterygoid, laterally and posterolaterally by the ectopterygoid, anterolaterally by the posterior portion of the palatal shelf of the maxilla, and medially by the palatine (Fig. 4C). The interpterygoid vacuity anteriorly represents the gap between the medial margins of the two palatines, and is gradually widening posteriorly following the laterally curved medial edges of the pterygoids. This opening is posteriorly edged by the basisphenoid, forming two basipterygoid processes, each of which obliquely contacts the corresponding pterygoid anteromedially (Fig. 4C).

In lateral view (Fig. 4B), the maxilla contacts the premaxilla, the septomaxilla and the nasal anteriorly, and further continues posteriorly by the jugal bone, constituting the posterior edge of the orbit, obliquely rising to contact the postorbital and postfrontal bones at its dorso-posterior tip. The anterior portion of the skull is dorsally connected to the posterior portion through the bones of skull roof and the postfrontal, postorbital and squamosal; while ventrally it is connected to the posterior portion through the pterygoids (Fig. 4B).

The epipterygoids well-developed as straight oblique columns extending from the fossae columellae on the antero-dorsal surface of each pterygoid in postero-dorsal direction where they contact the ventral processes of the parietals and approach but not contact the alar processes of the prootics (Fig. 4B). Stapes short, well-ossified (Fig. 4B); the quadrates are gently curved around the stapes, dorsally contacting the squamosal bone, medially contacting the lateral sides of the neurocranium. and posterodorsally contacting the supratemporal. Orbitosphenoids represented a pair of well-ossified small sickle-shaped bones located in the interorbital septum, visible in lateral view of the cranium (Fig. 4B).

Each mandible is comprised of two parts–the anterior portion is represented by the dentary, and is almost two times longer than the posterior portion of the mandible, formed by the bones of the articular complex. Each dentary with a dorsally elevated coronoid process, which is clearly visible in lateral view and matches the corresponding coronoid (Fig. 4B). The posterior part of the mandible, corresponding to the fused bones of the articular complex, is slightly slanted medially being almost parallel to the body axis in ventral view (Fig. 4C).

The hyoid apparatus is positioned ventrally to the neurocranium and has no direct contact with other skeletal elements (Fig. 4B). The hyoid apparatus comprises anteriorly a anterodorsally oriented, unpaired basihyal element fused to the long rapier-shaped processus lingualis. The basihyal is followed posteriorly by a pair of the first ceratobranchials in form of two laterally curved slender elements (Fig. 4B).

The pleurodont teeth appear to be bicuspid and have little to no difference in size; they are evenly spaced and present on the premaxillae (four teeth on each bone), the maxillae (21 teeth on each bone), and the dentaries (25 teeth on the left, and 27 teeth on the right dentary, respectively).

**Variation:** The paratypes match the holotype in most regards except for morphometric and meristic details outlined in Table 2. Coloration of the members of the type series in life showed no variation.

**Figure 8:**
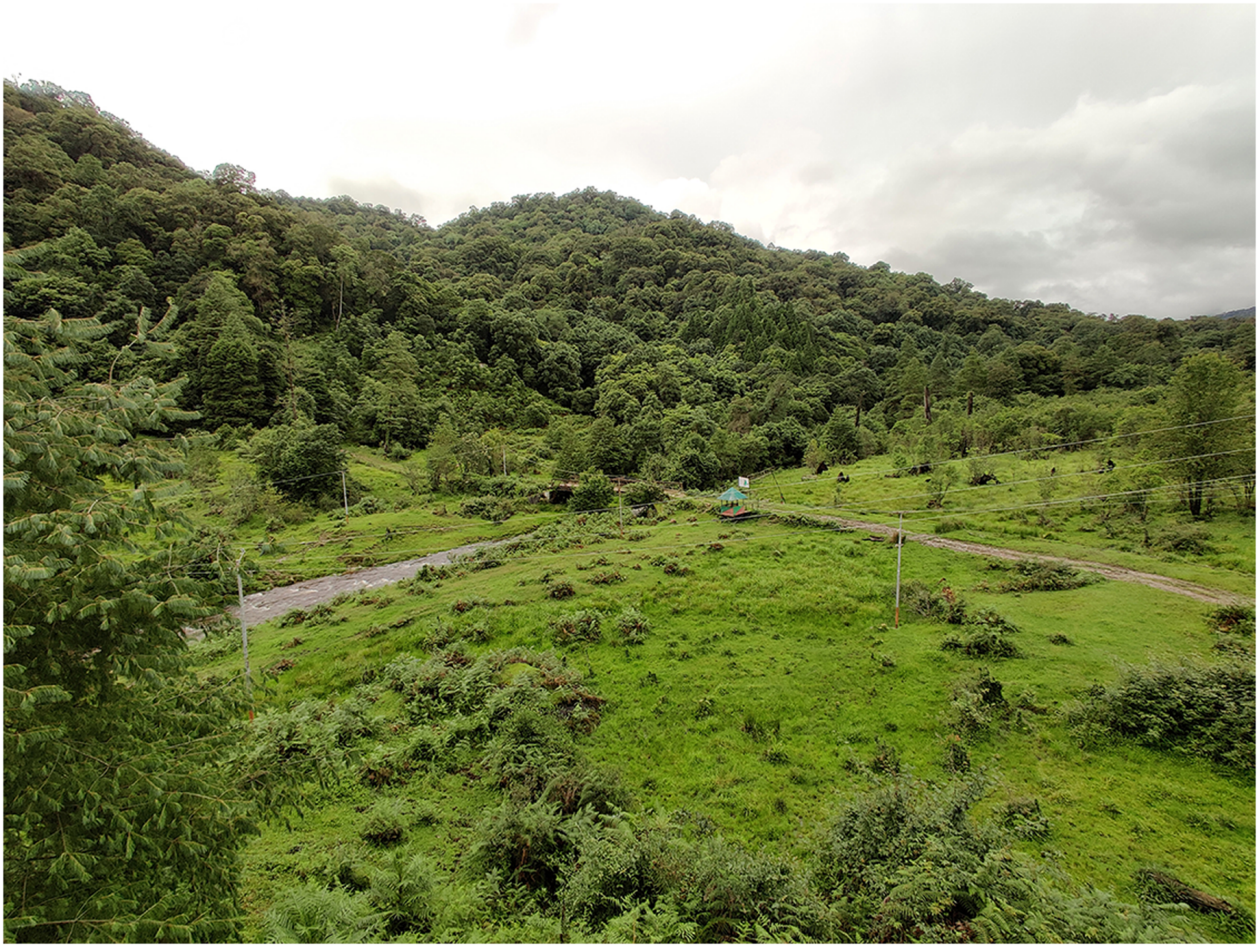
A general view of the *Protoblepharus apatani* gen. et sp. nov. habitat at the type locality in Pange camp, Talle Valley Wildlife Sanctuary. Photo by Zeeshan A. Mirza.

**Natural history and distribution:** The new species is currently known exclusively from Talle Valley Wildlife Sanctuary, East Kameng District, Arunachal Pradesh State of India (Fig. 2). The new species inhabits montane evergreen forests on elevations 1,800–2,000 m a.s.l., where it was usually recoded at forest clearings (Fig. 8). The forest type at the collection site is subtropical broad-leaved, temperate broad leaved, and temperate conifer forest types 8B/C1 and East Himalayan subtropical forest 11B/C (Champion & Seth, 1968). The members of the type series were collected in July; usually all individuals were recorded while seeking refuge under fallen logs. No other lizard taxa were found in sympatry, however, several snake species, including *Trachischium apteii* Bhosale, Gowande & Mirza, 2019, *Lycodon septentrionalis* (Günther, 1875), *Lycodon gammiei* (Blanford, 1878), *Ovophis monticola* (Günther, 1864) and *Pseudoxenodon macrops* (Blyth, 1855) were recorded in the same habitat; some of these species may represent predators of the new species. Other aspects of the *Protoblepharus apatani* **sp. nov.** ecology, including its diet and reproductive biology, remain largely unknown.

## Discussion

Phylogenetic relationships within the family Scincidae remain poorly resolved in the most recent phylogenetic studies (*e.g*., Pyron, Burbrink & Wiens, 2013; Linkem, 2013). This is especially true for many Asian groups of skinks, where recent progress in molecular phylogenetics have revealed numerous cases of paraphyly of many at genus level that, which were previously considered as well-established based on the morphological data (*e.g*., Honda et al., 2000, 2003; Linkem, Diesmos & Brown, 2011; Karin et al., 2016; Grismer et al., 2018, 2019; Poyarkov et al., 2019). In many cases such long-standing questions on generic taxonomy in Asian skinks remain unaddressed for a long time pending further progress in both taxon and gene sampling (Grismer et al., 2019). With the exception of certain genera, the lack of molecular data and more importantly, morphological data for correctly identified vouchered specimens, makes assessing phylogenetic relationships of many Asian skinks difficult and unreliable. In the present work we provide new insights on phylogenetic relationships and the systematics of the ablepharine skinks. Based on the new phylogenetic data we revise the taxonomic status and discuss relationships within the genera *Ablepharus*, *Asymblepharus* and *Himalblepharus*, and describe a new genus within this group, *Protoblepharus* **gen. nov**. Though this is not the first phylogenetic study on the ablepharine skinks, the previous studies could not address the questions of the basal radiation within the group due to the lack of specimens from the Himalayan and Central Asian regions (*e.g.*, Poulakakis et al., 2005; Skourtanioti et al., 2016; Xu et al., 2020). The sister relationships between the *Protoblepharus* **gen. nov.** and *Ablepharus sensu* lato, as well as the suggested distant position of several Himalayan lineages within the *Ablepharus* radiation, suggest an east Himalayan origin of the clade and, likely, an east to west pattern of diversification. This hypothesis must be further tested denser sampling across the range of the genus *Ablepharus*, including many species which remain unstudied from molecular perspective. A recent analysis by Xu et al. (2020) demonstrated that the uplift of the Himalaya played a key role in shaping the diversity of Asian herpetofauna; further studies are needed to shed light on the role of Himalaya in origin, and the spatio-temporal diversification of the ablepharine skinks.

The origin of a so-called “ablepharine eye” with partial or a complete fusion of the eyelids and formation of a transparent window in the lower eyelid, has been shown to have evolved multiple times across different scincid genera (*e.g.*, Fuhn, 1969; Greer, 1974; Datta-Roy et al., 2013). This is further reiterated by the findings in the present work wherein the taxa earlier identified as *Himalblepharus* **syn. nov.** (bearing a movable eyelid) and *Asymblepharus* **syn. nov.** (with a partial fusion of eyelids) are embedded within *Ablepharus*, which lack a movable eyelid. Further phylogenetic studies and a broader taxonomic sampling across the ablepharine lizards are needed to address the questions whether the *Ablepharus* taxa with the complete fusion of eyelids form a monophyletic group, and the factors that might shape the eyelid evolution in ablepharine skinks.

At present *Protoblepharus* **gen. nov.** is known from three isolated mountain areas in the Indian and Chinese portions of Eastern Himalaya (Fig. 2). Though all these localities belong to the southern slopes of Eastern Himalaya, they correspond to different isolated mountain valleys. The new genus, however, is expected be more widespread than is currently known. For example, Athreya (2006) and Agarwal, Mistry & Athreya (2010) reported *Asymblepharus sikimmensis* from Bompu and Lama camps, Eaglenest Wildlife Sanctuary, Arunachal Pradesh State. Scalation data and photographs presented therein well-match with that of the new genus. However, molecular data and a detailed examination of specimens from this locality would be necessary to confirm if they really belong to *Protoblepharus* **gen. nov.**, as well as to confirm their species identification. Furthermore, Deuti et al. (2020) reported the specimens of *Sphenomorphus apalpebratus* Datta-Roy, Das, Bauer, Lyngdoh-Tron & Karanth, 2013 from Talle Valley Wildlife Sanctuary based on the following series of specimens ZSI 26373–26377. During the survey conducted for the present work, the only scincid lizard we were able to record were the specimens of *Protoblepharus apatani* **gen. et sp. nov**. It would be necessary to ascertain if an additional scincid lizard taxa can be found at the type locality of the new species or if the specimens reported by Deuti et al. (2020) are actually conspecific with *Protoblepharus apatani* **gen. et sp. nov**.

Arunachal Pradesh by far remains the least explored Indian state in regard to its reptilian diversity, as *Protoblepharus apatani* **gen. et sp. nov**. described in the present work represents the fifth new reptile species discovered and described from a single rapid survey (see Bhosale, Gowande & Mirza, 2019; Bhosale et al., 2020; Mirza et al., 2020, 2021). Additionally four species of snakes were described from other areas in Arunachal Pradesh just in the last 2 years (Captain et al., 2019; Purkayastha & David, 2019; Das et al., 2020). Further intensified survey efforts are required to document the reptilian fauna of the region and to elaborate the corresponding conservation measures. Mountain forests in Arunachal Pradesh are threatened to a greater degree than in other parts of northeastern India (pers. obs.) and hence immediate efforts to document biodiversity of the region are imperative to ensure its conservation.

### Key to species of the genus *Protoblepharus* gen. nov.

1a. Nuchal scales present, lamellae on IV toe >11 2
1b. Nuchal scales absent, lamellae on IV toe 11 *P. medogensis* **comb. nov.**
2a. anterior border of tympanum with 1–7 projecting lobules, lamellae on IV toe 15 (rarely 16) *P. nyingchiensis* **comb. nov.**
2b. anterior border of tympanum lacking projecting lobules, lamellae on IV toe 12 (rarely 13) *Protoblepharus apatani* **sp. nov.**

## Conclusions

In this study, we present an updated multilocus phylogeny for the ablepharine skinks, which allow us to revise the taxonomy of the group and provide new insights on its origin and evolution. We report on a previously unknown deeply divergent clade of ablepharine skinks, inhabiting the Eastern Himalayan region, which we describe as a new genus; we further describe a new species of this clade from the Arunachal Pradesh State of India. The East Himalayan clade, described as *Protoblepharus* **gen. nov.**, forms a sister lineage with respect to all other ablepharine skinks, suggesting that the Eastern Himalaya might be the possible area of origin for the whole group. Within the remaining ablepharine skinks, we report that the presently adopted taxonomy, recognizing two genera with a complete fusion of eyelids (*Ablepharus sensu* stricto) and partial fusion of eyelids (*Asymblepharus* **syn. nov.**), is inaccurate and misleading, as the species with the completely fused eyelids are nested within the radiation of taxa with free or partially fused eyelids thus rendering the genus *Asymblepharus* **syn. nov.** paraphyletic. We propose to treat this group as a single genus *Ablepharus sensu* lato, and regard *Asymblepharus* **syn. nov.** and *Himalblepharus* **syn. nov.** as its subjective junior synonyms. Further studies on phylogenetic relationships of ablepharine skinks are needed to clarify the spatio-temporal process of their diversification, to test the ‘out of Himalaya’ biogeographic hypothesis, and to provide further insights into morphological evolution of this poorly studied group of lizards.

## Supplemental Information

10.7717/peerj.12800/supp-1 Supplemental Information 1 Maximum Likelihood phylogeny tree for selected scincid genera based on RAG1 gene.

10.7717/peerj.12800/supp-2 Supplemental Information 2 Left lateral view of the head and the anterior portion of the trunk of the holotype of *Protoblepharus apatani* gen. et sp. nov. (BNHS 2853) with the osteoderm cover not removed (visualized with CTVox and Amira 6.7). The position of the ear opening can be seen posterior to the mouth angle.

10.7717/peerj.12800/supp-3 Supplemental Information 3 Genbank accession numbers for samples of skinks used in the study.

10.7717/peerj.12800/supp-4 Supplemental Information 4 Sequence substitution model used for ML phylogeny.

10.7717/peerj.12800/supp-5 Supplemental Information 5 Sequences of skinks.

## Additional Information and Declarations

### Field Study Permissions

The following information was supplied relating to field study approvals (*i.e*., approving body and any reference numbers): The Department of Environment and Forest, Government of Arunachal Pradesh (permit nos. CWL/Gen/173/2018-19/Pt.V11/2421-33 to GG and CWL/Gen/173/2018-19/Pt.V11/2434-43 to ZAM).

### DNA Deposition

The following information was supplied regarding the deposition of DNA sequences: The sequences obtained in this study are available at GenBank: MZ820236–MZ820289, MZ827906–MZ827909, MZ790564–MZ790576, MZ790577–MZ790586, MZ790598–MZ790602.

### Data Availability

The following information was supplied regarding data availability: The raw morphological data are summarized in the Supplemental Files. The raw data is available as voucher specimens and tissue samples stored in the following herpetological collections: (1) BNHS, the Bombay Natural History Society, Mumbai, India; (2) NCBS, the collection of the National Centre for Biological Sciences, Bangalore, India; (3) ZMMU: Zoological Museum of Moscow University, Moscow, Russia. The microCT scan of the holotype is available at MorphoSource: DOI 10.17602/M2/M381781.

### New Species Registration

The following information was supplied regarding the registration of a newly described species: Publication LSID: urn:lsid:zoobank.org:pub:BE943DD6-3FBD-4A1B-B967-DCC2D9DAA175. Genus name: *Protoblepharus* urn:lsid:zoobank.org:act:2FF9E503-7E77-42E3-85EA-521CD3F260BC. Species name: *Protoblepharus apatani* urn:lsid:zoobank.org:act:A0776D1F-1399-47EC-8A10-96CE76E56290.
