# Supplementary figures and images for: A new ancient lineage of ablepharine skinks (Sauria: Scincidae) from eastern Himalayas with notes on origin and systematics of the group
Source: PeerJ. 2022 Jan 18;10:e12800. doi: 10.7717/peerj.12800 (PMC8781319; doi:10.7717/peerj.12800)

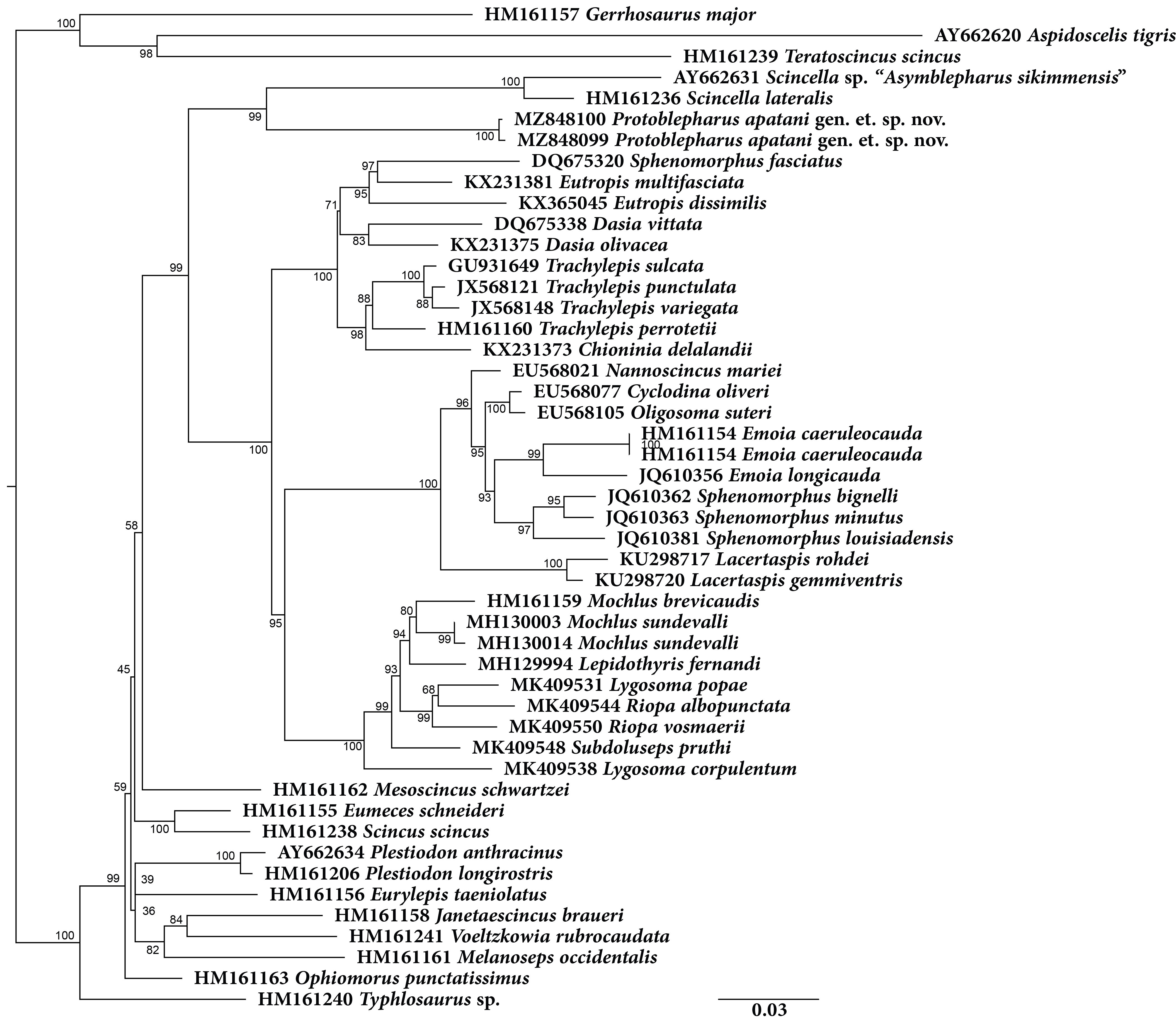

Supplement: Supplemental Information 1 [file peerj-10-12800-s001.jpg]

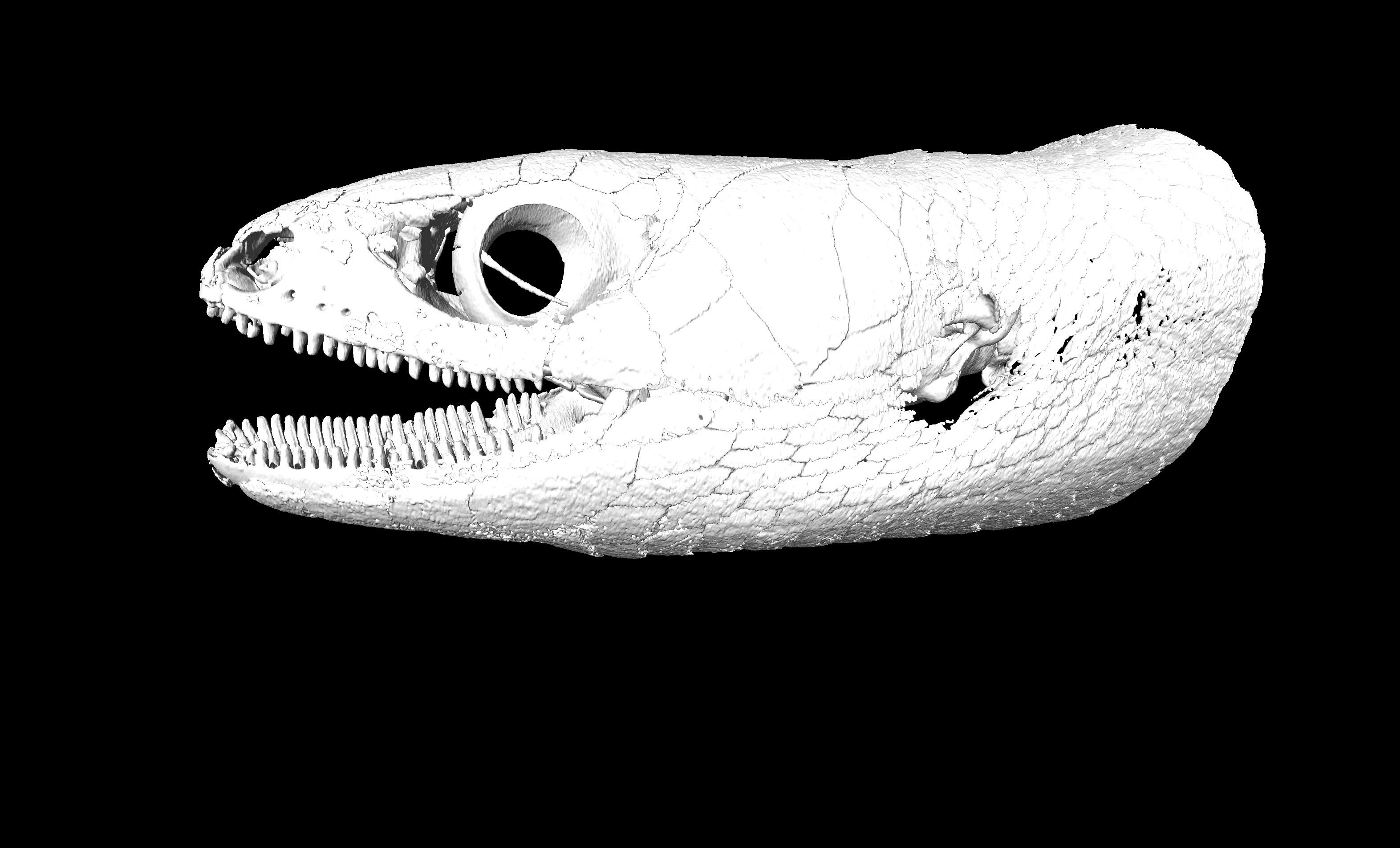

Supplement: Supplemental Information 2 — The position of the ear opening can be seen posterior to the mouth angle. [file peerj-10-12800-s002.jpg]
