# Supplementary material for: A new ancient lineage of ablepharine skinks (Sauria: Scincidae) from eastern Himalayas with notes on origin and systematics of the group
Source: PeerJ. 2022 Jan 18;10:e12800. doi: 10.7717/peerj.12800 (PMC8781319; doi:10.7717/peerj.12800)
Supplement: Supplemental Information 4 [file peerj-10-12800-s004.docx]

Supplementary Table S2. Sequence substitution model used for phylogenetic analysis

ML phylogeny based on Rag1

| Gene | Codon position | Model |
| --- | --- | --- |
| Rag1 | Rag1_1 | HKY+F+G4 |
|  | Rag1_2 | TPM2+F+G4 |
|  | Rag1_3 | TVMe+G4 |

Concatinated dataset ML phylogeny

| Gene | Codon position | Model |
| --- | --- | --- |
| *ND*2 | nd2_codon1 | HKY+F+I+G4 |
|  | nd2_codon2 | GTR+F+I+G4 |
|  | nd2_codon3 | TPM2u+F+G4 |
| *Cyt* b | cytb_codon1 | TIMe+I+G4 |
|  | cytb_codon2 | GTR+F+G4 |
|  | cytb_codon3 | TIM2e+I+G4 |
| 16S | - | TIM2+F+I+G4 |
| 12S | - | TIM2+F+I+G4 |
| *Mc1r* | mc1r_codon1 | HKY+F+G4 |
|  | mc1r_codon2 | JC |
|  | mc1r_codon3 | F81+F |
| Nktr | nktr_codon1 | TN+F+I |
|  | nktr_codon2 | K2P+G4 |
|  | nktr_codon3 | K3P+G4 |

Concatinated dataset BI phylogeny

| Gene | Codon position | Model |
| --- | --- | --- |
| *ND*2 | nd2_codon1 | HKY+I+G |
|  | nd2_codon2 | GTR+ I+G |
|  | nd2_codon3 | GTR+ I+G |
| *Cyt* b | cytb_codon1 | GTR+ I+G |
|  | cytb_codon2 | GTR+G |
|  | cytb_codon3 | SYM+I+G |
| 16S | - | GTR+ I+G |
| 12S | - | GTR+ I+G |
| *Mc1r* | mc1r_codon1 | HKY+G |
|  | mc1r_codon2 | JC |
|  | mc1r_codon3 | F81 |
| Nktr | nktr_codon1 | GTR+G |
|  | nktr_codon2 | GTR+G |
|  | nktr_codon3 | K80+G |
