# Supplementary material for: A new ancient lineage of ablepharine skinks (Sauria: Scincidae) from eastern Himalayas with notes on origin and systematics of the group
Source: PeerJ. 2022 Jan 18;10:e12800. doi: 10.7717/peerj.12800 (PMC8781319; doi:10.7717/peerj.12800)
Supplement: Supplemental Information 5 [file peerj-10-12800-s005.pdf]

SEQUENCES OF SKINKS
NEWLY GENERATED FOR THE PRESENT STUDY

(NB: For each sequence the GenBank Accession Number, museum voucher ID and the species name is provided, for details see Supplementary Table S1).

>NHMC80.3.131.57_Ablepharus_anatolicus
ATTGTCCGCCAGAGAACTACGAGCGTAAAACTTAAA
ACTCCAAGGACTTGGCGGTGCCCCACACCAACCTAGAGGAGCCTGTCCTATAATCGATAC
TCCACGT-TCAACCTTACCCCCCCTAGT-AAACCCAGCCTATATACCGCCGTCGACAGCC
CACCTTATGA-AAGAAA-CCAAGTGAGCACAATAATTAACAACTAATACGTCAGGTCAAG
GTGTAGCACACGG-GTGGGCAGAGATGGGCTACATTCCCTCT-AACAGGAAACACGCACA
GTGTGTTGAAAC-ACACACTTAAAGGCGGATTTAGCAGTAAGACAGATAA-GAA--TACC
TGTCTC-AAACTTGCTCTGGGGCGCGCACACACCGCCCGT-

>NHMC80.3.131.8_Ablepharus_budaki
ACTGTTCGCCAGAGAACTACGAGCGAAAAACTTAAA
ACTCTAAGGACTTGGCGGTGCCCCATACCAACCTAGAGGAGCCTGTCCTATAATCGATAT
TCCGCGC-TAAACCTCACCCCCCCTTGT-AAACCCAGCCTATATACCGCCGTCGACAGCT
CACCTTATGA-AAGAAT-CCAAGTGAGCATAATAGTTAACAACCAATACGTCAGGTCAAG
GTGTAGCACACGG-GGGGACAGAGATGGGCTACATTTCCTAC-AATAGGAAACACGCACG
GCACGCTGAAAC-ATGCGCCCAAAGGTGGATTTAGCAGTAAGACAGATAA-GAA--GGCC
TGTCTCAAAACTTGCTCTGGGGCGCGCACACACCGCCCGT-

>BAM-36_Ablepharus_deserti
ATTGTTCGCCAGAGAACTACAAGCGAAAAACTTAAA
ACTCCAAGGACTTGGCGGTGCCCCACACCAACCTAGAGGAGCCTGTCCTATAATTGATAC
TCCACAC-TAAACCTTACCCCCCCTTGA-TAACCCAGCCTATATACCGCCGTCGCCAGCC
TACCTTGTGA-AAGGAG-CCCAGTGGGCACAATAATTAACAATTAACACGTCAGGTCAAG
GTGTAGTACATGG-GGCGGTAGAGATGGGCTACATTTCCTTT-AACAGGAGACACGCACA
GTGGGTTGAAAC-GCCCACTTAAAGGTGGATTTAGCAGTAAGACATGTAA-GAA--ACCA
TGTCTC-AAACCTGCTCTGGGACGCGCACACACCGCCCGT-

>R-14554_Ablepharus_pannonicus
ACTGTTCGCCAGAGAACTACGAGCGGAAAACTTAAA
ACTCCAAGGACTTGGCGGTGCCCCACACCAACCTAGAGGAGCCTGTCCTATAATCGATAC
TCCACGC-TTAACCTTACCCACCCTTG--AAACTCAGCCTATATACCGCCGTCGACAGCC
TACCTTGTGA-AAGTAA-ATAAGTTGGCACAATAGTTAATAACTAACACGTCAGGTCAAG
GTGTAGCACATGG-GCGGGTAGAGATGGGCTACATTTCCTAA-TATAGGAAACACGCACA
GTGCAATGAAAT-ATGCACCTTAAGGTGGATTTAGCAGTAAGACAAACAA-GAACCCCCC
TGCCTA-AAACCTGCTCTGGGGCGCGCACACACCGCCCGT-

>BAM-1111_Ablepharus_kitaebelii
ATTGTTCGCCAGAGAACTACAAGTGAAAAACTTAAA
ACTCCAAGGACTTGGCGGTGCCCCATATCAACCTAGAGGAGCCTGTCCTATAATCGATAC
TCCACGC-TTAACCCCACCCCCTTTTG--AAATACAGCCTATATACCGCCGTCGCCAGCC
CACCTTATGA-AAGAAA-TTCAGTGGGCACAATAGTTAACAACTAATACGTCAGGTCAAG
GTGTAGCACATGA-GGGGGCAGAGATGGGCTACATTTCCTAC-AACAGG-AACACGCACG
GCATATTGAAAT-ATATACCCCAAGGCGGATTTAGCAGTAAGACAAACAA-GAC--AAAC
TGTCTT-AAACCTGCTCTGGGACGCGCACACACCGCCCGT-

>NHMC80.3.82.2_Ablepharus_kitaibelii
ATTGTTCGCCAGAGAACTACGAGTGAAAAACTTAAA
ACTCTAAGGACTTGGCGGTGCCCCATATCAACCTAGAGGAGCCTGTCCTATAATCGATAC

TCCACGC-TTAACCTTACCCCCTTTTG--AAACACAGCCTATATACCGCCGTCGCCAGCC
CACCTTATGA-AAGAAA-TTCAGTGGGCATAATAGTTAACAACTAATACGTCAGGTCAAG
GTGTAGCACATGG-GGGGGCAGAGATGGGCTACATTTCCTCC-AACAGGAAACACGCACG
GCACACTGAAAT-ATATGCCCTAAGGCGGATTTAGCAGTAAGACAAACAA-GAC-AAACC
TGTCTT-AAACCTGCTCTGGGACGCGCACACACCGCCCGT-

>NHMC80.3.169.5_Ablepharus_rueppellii
ACTGTTCGCCAGAGAACTACGAGCGAAAAACTTAAA
ACTCTAAGGACTTGGCGGTGCCCCACACCAGCCTAGAGGAGCCTGTCCTATAATCGATAC
TCCGCGT-TAAACCTTACCCCCTCTTGT-AAACCCAGCCTATATACCGCCGTCGACAGCT
CACCTTGTGA-AAGACA-CTTAGTGGGCACAACAATTATCAACTAATACGTCAGGTCAAG
GTGTAGCACACGA-GGGGGCAGAGATGGGCTACATTTCCTCT-AACAGGAAACACGCACG
ACATGCTGAAAC-ACATGCCCAAAGGCGGATTTAGCAGTAAGACAGATAA-GAA--GACC
TGTCTC-AAACCTGCTCTGGGGCGCGCACACACCGCCCGT-

>BAM-15_Asymblepharus_eremchenkoi
GCTGTCCGCCTGAGAACTACAAGCGTAAAGCTTAAA
ACTCCAAGGACTTGGCGGTGCTCCATATCAACCTAGAGGAGCCTGTCCTATAATCGATAC
TCCACGC-TCAACCTTACCCCCTCTTG--AAACCCAGCCTATATACCGCCGTCACCAGCC
CACCTTATGA-GAGAAT-TTAAGTGAGCACAATAATTATTAACTAACACGTCAGGTCAAG
GTGTAGCACACGC-GGGGGCAGAGATGGGCTACATTTCCTTA-TACAGGAAACACGAACA
GTGTTCTGAAAC-ACCCACTCGAAGGCGGATTTAGCAGTAAGACAAATAA-GAA--CCCT
TGTCTA-AAACCTGCTCTGGAGCGCGCACACACCGCCCGT-

>BAM-135_Asymblepharus_alaicus
ATTGTTCGCCTGAGAACTACAAGCGTAAAACTTAAA
ACTCCAAGGACTTGGCGGTGCTCCATATCAACCTAGAGGAGCCTGTCCTATAATCGATAC
TCCACGC-TTAACCTTACCCCCCCTTG--AAACCCAGCCTATATACCGCCGTCACCAGCC
CACCTTATGA-AAGAAG-TTAAGTGAGCACAACAATTATAAACTAACACGTCAGGTCAAG
GTGTAGCACACGC-GGGGGCAGAGATGGGCTACATTTCCTTC-TA-AGGAAACACGAACA
GTGTTATGAAAC-ACCCACTCGAAGGCGGATTTAGCAGTAAGACAAATAA-GAA---TCC
TGTCTC-AAACCCGCTCTGGAGCGCGCACACACCGCCCGT-

>KIZ015959_Asymblepharus_ladacensis
ACTATTCGCCAGAGAACTACAAGCGAAAAACTTTAA
ACTCCAAGGACTTGGCGGTGCTCCACACCAGCCTAGAGGAGCCTGTCCTATAATCGATAT
TCCACGC-TCAACCTTACCCCCCCTTG--AAACTCAGCCTATATACCGCCGTCGCCAGCC
TACCTTATGA-AAG-GA-TAAAGTGAGCACAATAGTTAACAACTAATACGTCAGGTCAAG
GTGTAGCACACGG-G--GGGAGAGATGGGCTACATTCCCTCC-CACAGGGAA-ACGAACA
GTGTATTGAAAT-ACACACTCAAAGGTGGATTTAGCAGTAAAACAAATTA-TAC--CCCT
TTATTT-CAATATGCTCTGGAGCGCGCACACACCGCCCGT-

>NAP-9870_Asymblepharus_nepalensis
ACTGTTCGCCAGAGAACTACAAGCGAAAAACTTAAA
ACTCCAAGGACTTGGCGGTACTCCACATCAACCTAGAGGAGCCTGTCCTATAATCGATAC
TCCACGT-TAAACCTTACCACCCCTTG--AAACACAGCCTATATACCGCCGTCGACAGCC
CACCTTATGA-AAGACA-CCAAGTGAGCACAATAGCTAGCAGTTAATACGTCAGGTCAAG
GTGTAGCACACGG-GGTGGCAGCGATGGGCTACATTTCCTAC-AACAGGACATACGAACG
GCATACTGAAAT-ACCCACCTAAAGGCGGATTTAGCAGTAAAACAAATAA-GAG--TTAT
TGTTTT-AAACCTGCTCTGGAGTGCGCACACACCGCCCGT-

>NAP-9807_Asymblepharus_mahabharatus
ACTGTTCGCCAGAGAACTACAAGCGAAAAACTTAAA
ACTCTAAGGACTTGGCGGTGCTCCATACCAACCTAGAGGAGCCTGTCCTATAATCGATAC

CCCACGC-TCAACCTTACCCCCCCCTAA-AAACACAGCCAATATACCGCCTTCCCCAGCC
TACCTAATGA-AAAAAA-TTAGTTGAGCACAATAATTAACAACTAGCACTTCAGGTCAAG
GGGTGCCAAAGGG-GGCGGCAAAAATGGGCTACTTTTCCTCC-TACAGGAAATACTTACA
GGGTAAGGAAAC-ACCCACCTTAAGGGGGATTTACCATTAAACCAACCTA-CCA--TTAT
GGTTTC-AAACCGGCTCTGGAGCGCGCACACCCCGCCCGC-

>AY607282_Asymblepharus_mahabharatus
ACTGTTCGCCAGAGAACTACAAGCGAAAAACTTAAA
ACTCTAAGGACTTGGCGGTGCTCCATACCAACCTAGAGGAGCCTGTCCTATAATCGATAC
CCCACGC-TCAACCTTACCCCCCCCTAA-AAACACAGCCAATATACCGCCTTCCCCAGCC
TACCTAATGA-AAAAAA-TTAGTTGAGCACAATAATTAACAACTAGCACTTCAGGTCAAG
GGGTGCCAAAGGG-GGCGGCAAAAATGGGCTACTTTTCCTCC-TACAGGAAATACTTACA
GGGTAAGGAAAC-ACCCACCTTAAGGGGGATTTACCATTAAACCAACCTA-CCA--TTAT
GGTTTC-AAACCGGCTCTGGAGCGCGCACACCCCGCCCGC-

>NAP-9840_Asymblepharus_sikkimensis_2
ACTGTTCGCCCGAGAACTACAAGCGAAAAACTTAAA
ACTCTAAGGACTTGGCGGTGCTCCATATCAACCTAGAGGAGCCTGTCCTATAATCGATAC
CCCACGC-TACACCTCACCACCCCTAG--AAACCCAGCCTATATACCGCCGTCGACAGCC
TACCTTATGA-GAGAAA-TTCAGTAGGCACAACAATTAGTAACTAATACGTCAGGTCAAG
GTGTAGCATATGG-GGTGGTAGAGATGGGCTACATTTCCTCC-AACAGGAAATACGCACA
GTGTACTGAAAC-ACCCACCCAAAGGCGGATTTAGCAGTAAGACAAATAA-GCA--TTAT
TGCCTC-AAACCTGCTCTGGAGCGCGCACACACCGCCCGT-

>NAP-9816_Asymblepharus_sikkimensis_3
ACTGTTCGCCCGAGAACTACAAGCGAAAAACTTAAA
ACTCTAAGGACTTGGCGGTGCTCCATATCAACCTAGAGGAGCCTGTCCTATAATCGATAC
CCCACGC-TAAACCTCACCCCCCCTAG--AAACCCAGCCTATATACCGCCGTCGACAGCC
TACCTTATGA-AAGAAA-TTCAGTAGGCACAACAATTAGCAACTAATACGTCAGGTCAAG
GTGTAGCATATGG-GGTGGTAGAGATGGGCTACATTTCCTAC-AACAGGAAATACGCACA
GTGTACTGAAAT-ACCCACCCAAAGGCGGATTTAGCAGTAAGACAAATAA-GCA--TTAT
TGCCTC-AAACCTGCTCTGGAGCGCGCACACACCGCCCGT-

>NAP-9858_Asymblepharus_sikkimensis_1
ACTGTTCGCCCGAGAACTACAAGCGAAAAACTTAAA
ACTCTAAGGACTTGGCGGTGCTCCATATCAACCTAGAGGAGCCTGTCCTATAATCGATAC
CCCACGC-TACACCTCACCCCCCCTAG--AAACCCAGCCTATATACCGCCGTCGACAGCC
TACCTTATGA-GAGAAA-TTCAGTAGGCACAACAATTAATAACTAATACGTCAGGTCAAG
GTGTAGCATATGG-GGTGGTAGAGATGGGCTACATTTCCTCC-AACAGGAAATACGCACA
GTGTACTGAAAC-ACCCACCCAAAGGCGGATTTAGCAGTAAGACAAATAA-GCA--TTAT
TGTCTC-AAACCTGCTCTGGAGCGCGCACACACCGCCCGT-

>ZM146_Protoblepharus_sp.nov.
AAAAAAAAACAGTAAAACCACAGTACTGTTCGCCAGAGAACTACAAGCGAAAAACTTAAA
ACTCTAAGGACTTGGCGGTGCTCCACACCGACCTAGAGGAGCCTGTCCTACAATCGATAC
CCCACGT-TCAACCTCACCCCCTCTTG--AAAACCAGCCTATATACCGCCGTCGACAGCC
TACCTTATGA-AAGAAA-TAAAGTGAGCATAACAGTTATTAACTAGTACGTCAGGTCAAG
GTGTAGCAAACGA-GGCGGCAGAGATGGGCTACATTTCCTAC-CACAGGAAAAACGAACA
GCGTGCTGAAAC-ATACACTTGAAGGCGGATTTAGCAGTAAGATAAACAA-GAG--ATGC
TATCTA-A-

>BAM-36_Ablepharus_deserti
---GCCCAGTGAAA--TATTTCAACGGCCGCGG-TATTCTAACCGTGCAAAGGTAGCGTAATCA
CTTGTCTTCCAAATAAAGACCAGTATGAA-CGGCTAAATGAGGACAAACCTGTCTCCTGC

GACCGATCAGTGAAATTGATCTTCTGGTGCAAAAGCCAGAATAATCACATAAGACGAGAA
GACCCTGTGGAGCTTAAAATCTAT-TATTACCTAAA-ACTAATATTAATTTTAAGTTGGG
GCGACTTCGGAATAAAACAAAACTTCCGAGCAAGGA-GAC-ATA-T---AC-TCCA-AA-
TA-AGGCCCACAAGCC-AT---TAGCCGACCCAGT-CACACT--GATCAACGAACC
AAGTTACCCCAGGGATAACAGCGCAATCCCCTTCAAGAGTCCATATCGACAAGGGGGTTT
ACGACCTCGATGTTGGATCAGGACACCCCAATGGTGCAACCGCTATTAAAGGTTCGTTTG
TTCAACGATTAATAGTCCTACGTGATCTG

>BAM-1111_Ablepharus_kitaebelii
---GCCCA
GTGAAA--CATTTTAACGGCCGCGG-TATTCTAACCGTGCAAAGGTAGCGTAATCA
CTTGTCTTCCAAATAGAGACCAGTATGAA-TGGCTAAATGAGGGCAAACCTGTCTCCTGC
ACCCAATCAGTGAAATTGATCTCCTGGTACAAAAGCCAGAATAATTACATAAGACGAGAA
GACCCTGTGGAGCTTAAAATTTAT-AACTACC-AAA-ACTAGTATTAATTTTAAGTTGGG
GCGACTTCGGAACAAAACAAAACTTCCGAGCTAGGA-GTT-ATT-T---AC-TCCT-AA-
TA-AGGCCGACAAGCC-AA---CAACCGACCCAGT-CACACT--GATCAACGAACC
AAGTTACCCCAGGGATAACAGCGCAATCCCCTCCAAGAGTCCGTATCGACAAGGGGGTTT
ACGACCTCGATGTTGGATCAGGACACCCTAATGGTGCAGCCGCTATTAAAGGTTCGTTTG
TTCAACGATTAATAGTCCTACGTGATCTG

>R-14554_Ablepharus_pannonicus
---GCCCA
GTGAAG--TATTTTAACGGCCGCGG-TATTCTAACCGTGCAAAGGTAGCGTAATCA
CTTGTCTTCTAAATAAAGACCAGTATGAA-TGGCTAAATGAGGACAGACCTGTCTCCTGC
ACCCAATCAGTGAAATTGATCTTCTGGTCCAAAAGCCAGAATATTTCCACAAGACGAGAA
GACCCTGTGGAGCTTAAAATTTAA-TGCTACTTTAA-ATTAGCACTCATTTTAAGTTGGG
GCGACTTCGGAACAAAACAAAACTTCCGAGCAAGGA-GCC-ACC-C---AC-TCCT-AA-
TA-AGGCTTACACGCC-AA---CAACAGACCCAGT-CATACT--GATCAACGAACC
AAGTTACCCCAGGGATAACAGCGCAATCCCCTTCAAGAGTCCATATCGACAGGGGGGTTT
ACGACCTCGATGTTGGATCAGGACACCCAGATGGTGCAGCCGCTATCAAAGGTTCGTTTG
TTCAACGATTAATAGTCCTACGTGATCTG

>BAM-15_Asymblepharus_eremchenkoi
---GCCCA
GTGAAC--TATTTCAACGGCCGCGG-TATTCTAACCGTGCAAAGGTAGCGTAATCA
CTTGTCTTCTAAATAAAGACCAGTATGAA-TGGCTAAATGAGGGCAAACCTGTCTCCTGC
GCCCAATCAGTGAAACTGATCTTCTGGTACAAAAGCCAGAATAATAACATAAGACGAGAA
GACCCTGTGGAGCTTGAAACTTAT-GCCTACATAAA-CCTAGGCCAAGTTTTAAGTTGGG
GCGACTTCGGAATAAAACAACACTTCCGAGCAAGGG-ACT-ATC-A---GTCTCCA-AA-
TA-AGGCCCACAAGCC-GAC--TAGCCGACCCAGT-CATACT--GATCAACGAACC
AAGTTACCCCAGGGATAACAGCGCAATCCCCCTCAAGAGTCCCTATCGACAGGGGGGTTT
ACGACCTCGATGTTGGATCAGGACACCCAAATGGTGCAGCAGCTATTAAAGGTTCGTTTG
TTCAACGATTAACAGTCCTACGTGATCTG

>BAM-135_Asymblepharus_alaicus
---GCCCA
GTGAAC--TATTTCAACGGCCGCGG-TATTCTAACCGTGCAAAGGTAGCGTAATCA
CTTGTCTTCTAAATAAAGACCAGTATGAA-TGGCTAAATGAGGGCAAACCTGTCTCCTGC
GCCCAATCAGTGAAACTGATCTTCTGGTACAAAAGCCAGAATAATAACATAAGACGAGAA
GACCCTGTGGAGCTTGAAACTTAC-GCCTACATAAA-CCTAGGCCAAATTTTAAGTTGGG
GCGACTTCGGAATAAAACACCACTTCCGAGCAAGGA-GCT-ATA-A---ACCTCCA-AA-
CA-AGGCCCACAAGCC-GAC--TAGCCGACCCAGT-CATACT--GATCAACGAACC
AAGTTACCCCAGGGATAACAGCGCAATCCCCCTCAAGAGTCCATATCGACAGGGGGGTTT
ACGACCTCGATGTTGGATCAGGACCCCCAAATGGTGCAGCAGCTATTAAAGGTTCGTTTG

TTCAACGATTAATAGTCCTACGTGATCTG

>NAP-9807_Asymblepharus_mahabharatus

--TTTCAACGGCCGCGG-TATTCTAACCGTGCAAAGGTAGCGTAATCA
CTTGTCTTCTAAATAAAGACCAGTATGAA-TGGCTAAATGAGGACAAACCTGTCTCCTGC
ACCCAATCAGTGAAATTGATCTTCTAGTACAAAAGCTAGAATAATCACATAAGACGAGAA
GACCCTGTGGAGCTTAAAA-TTAT-GACTACCACAA-CCTAGTCTGAATTTTGAGTTGGG
GCGACTTCGGAACAAAATAAACCTTCCGAGCAAGGA-ATA-AAT---ATTCC-AA-
CA-AGGCAAACAAGCC-GA---CAGCCGACCCAGT-CACACT--GATCAACGAACC
AAGTTACCCCAGGGATAACAGCGCAATCCCCTTCAAGAGTCCCTATCGACAAGGGGGTTT
ACGACCTCGATGTTGGATCAGGACACCCCAATGGTGTAGCCGCTATTAATGGTTCGTTTG
TTCAACGATTAACAGTCCTACGTGATCTG

>NAP-9870_Asymblepharus_nepalensis

--TTTAAACGGCCGCGG-TATTCTAACCGTGCAAAGGTAGCGTAATCA
CTTGTCTTCTAAATAAAGACCAGTATGAA-TGGCTAAATGAGGACAAACCTGTCTCCTGT
GACCAATCAGTGAAACTGATCTTCCGGTACAAAAGCCGGAATAACCCCATAAGACGAGAA
GACCCTGTGGAGCTTAAAA-TTAT-AACTACCTAAA-ACTAGCACTAATTTTAAGTTGGG
GCGACTTCGGAACCAAATAAAACTTCCGAGCAAAGA-ACC-AGC-A-ATTCC-AA-
CA-AGGCCTACATGCC-AT---GAGCCGACCCAGT-CACACT--GATCAACGAACC
AAGTTACCCCAGGGATAACAGCGCAATCCCCCCCAAGAGTCCATATCGACGGGGGGGTTT
ACGACCTCGATGTTGGATCAGGACATCCCAATGGTGCAACCGCTATTAAAGGTTCGTTTG
TTCAACGATTAATAGTCCTACGTGATCTG

>NAP-9840_Asymblepharus_sikkimensis_2

--TTTAAACGGCCGCGG-TATTCTAACCGTGCAAAGGTAGCGTAATCA
CTTGTCTTCTAAATAAAGACCAGTATGAA-TGGCTAAATGAGGACAAACCTGTCTCCTGC
ACCCAATCAGTGAAACTGATCTTCCAGTACAAAAGCTGGAATAATCACATAAGACGAGAA
GACCCTGTGGAGCTTAAAATTTAT-AACTACTTTAA-CCTAGTACAAATTTTAAGTTGGG
GCGACTTCGGAACAAAATAAAACTTCCGAGCAAGGA-GTA-TTACTCC-AA-
CA-AGGCCTACAAGCC-GA---CAGCCGACCCAGT-CATACT--GACCAACGAACC
AAGTTACCCCAGGGATAACAGCGCAATCCCCTTCAAGAGTCCTTATCGACAAGGGGGTTT
ACGACCTCGATGTTGGATCAGGACACCCCAATGGTGCAGCCGCTATTAAAGGTTCGTTTG
TTCAACGATTAATAGTCCTACGTGATCTG

>NAP-9816_Asymblepharus_sikkimensis_3

--TTTAAACGGCCGCGG-TATTCTAACCGTGCAAAGGTAGCGTAATCA
CTTGTCTTCTAAATAAAGACCAGTATGAA-TGGCTAAATGAGGACAAACCTGTCTCCTGC
ACCCAATCAGTGAAACTGATCTTCCAGTACAAAAGCTGGAATAATCACATAAGACGAGAA
GACCCTGTGGAGCTTAAAATTTAT-GACTACTTTCA-CCTGGTACAAATTTTAAGTTGGG
GCGACTTCGGAACAAAATAAAACTTCCGAGCAAGGA-GCA-TTACTCC-AA-
CA-AGGCCTACAAGCC-AA---TAGCCGACCCAGT-CACACT--GACCGACGAACC
AAGTTACCCCAGGGATAACAGCGCAATCCCCTTCAAGAGTCCTTATCGACAGGGGGGTTT
ACGACCTCGATGTTGGATCAGGACACCCCAATGGTGCAGCCGCTATTAAAGGTTCGTTTG
TTCAACGATTAATAGTCCTACGTGATCTG

>NAP-9858_Asymblepharus_sikkimensis_1

--TTTAAACGGCCGCGG-TATTCTAACCGTGCAAAGGTAGCGTAATCA
CTTGTCTTCTAAATAAAGACCAGTATGAA-TGGCTAAATGAGGACAAACCTGTCTCCTGC

ACCCAATCAGTGAAACTGATCTTCCAGTACAAAAGCCGGAATAATCACATAAGACGAGAA
GACCCTGTGGAGCTTGAAATTTAT-AGCTACTTTAA-CCTAGTACAAATTTTAAGTTGGG
GCGACTTCGGAACAAAACAAAACTTCCGAGCAAGGA-GTA-TTACTCC-AA-
CA-AGGCCTACAAGCC-AA---TAGCCGACCCAGT-CATACT--GATCAACGAACC
AAGTTACCCCAGGGATAACAGCGCAATCCCCTTCAAGAGTCCTTATCGACAGGGGGGTTT
ACGACCTCGATGTTGGATCAGGACACCCCAATGGTGCAGCCGCTATTAAAGGTTCGTTTG
TTCAACGATTAACAGTCCTACGTGATCTG

>ZM146_Protoblepharus_sp.nov.
CGCCTGTTTATCAAAAACATAGCCTTTAGCAAAACAAGTATTAAAGGTCTCGCCTGCCCA
GTGAAA--CATTTAAACGGCCGCGG-TATTCTAACCGTGCAAAGGTAGCGTAATCA
CTTGTCTTCTAAATAAAGACCAGTATGAA-CGGCTAAATGAGGACAAATCTGTCTCCTGC
AACCAATCTGTGAAATTGATCTTCCCGTACAAAAGCTGGAATAACCACATAAGACGAGAA
GACCCTGTGGAGCTTAAAATCAAC-TGCTACACAAA-CTAGCATAAGATTTTAGGTTGGG
GCGACTTCGGAACAAAATAAAACTTCCGAGCACGGA-ACC-ACC-ACTTCC--AA-
AA-AGGCCTACGAGCC-AA---GCACTGACCCAGT-CACACT--GATCAACGAACC
AAGTTACCCCAGGGATAACAGCGCAATCCCCTTCAAGAGTCCATATCGACAAGGGGGTTT
ACGACCTCGATGTTGGATCAGGACACCCAAATGGTGCAGCCGCTA---
-
>ZM145_Protoblepharus_sp.nov.
--CCTGTTTTACAAAAACATAGCCTTTAGCAAAACAAGTATTAAAGGTCTCGCCTGCCCA
GTGAAA--CATTTAAACGGCCGCGG-TATTCTAACCGTGCAAAGGTAGCGTAATCA
CTTGTCTTCTAAATAAAGACCAGTATGAA-CGGCTAAATGAGGACAAATCTGTCTCCTGC
AACCAATCTGTGAAATTGATCTTCCCGTACAAAAGCTGGAATAACCACATAAGACGAGAA
GACCCTGTGGAGCTTAAAATCAAC-TGCTACACAAA-CTAGCATAAGATTTTAGGTTGGG
GCGACTTCGGAACAAAATAAAACTTCCGAGCACGGA-ACC-ACC-ACTTCC--AA-
AA-AGGCCTACGAGCC-AA---GCACTGACCCAGT-CACACT--GATCAACGAACC
AAGTTACCCCAGGGATAACAGCGCAATCCCCTTCAAGAGTCCATATCGACAAGGGGGTTT
ACGACCTCGATGTTGGATCAGGACACCCAAATGGTGCAGCCGCTA---
-
>NAP-6640_Scincella_melanosticta
---GCCCA
GTGAAA--TTTTTTAACGGCCGCGG-TATTCTAACCGTGCAAAGGTAGCGTAATCA
CTTGTCTTCTAAATAAAGACCAGTATGAA-CGGCTAAATGAGGATAAACCTGTCTCCTAC
AACTAATCAGTGAAACTGATCTTCCAGTCCAAAAGCTGGAATTCCAACACAAGACGAGAA
GACCCCGTGGAGCTTAAGACGAAC-CACTAAT--AT-ACTAGAAAAAGTCTTAAGTTGGG
GCGACTTCGGAACAAAATAAAACTTCCGAGCACAGA-GCC-ACA--C-CTCT-AA-
CTAAGGCTAACAAGCC-AAA--GAATTGACCCAGT-CATACT--GACTAACGAACC
AAGTTACCCCGGGGATAACAGCGCCATCTTCTTCAAGAGTCCCTATCGACAAGAAGGTTT
ACGACCTCGATGTTGGATCAGGACACCCAAATGGTGAAGCCGCTATTAAAGGTTCGTTTG
TTCAACGATTAACAGTCCTACGTGATCTG

>NAP-1331_Scincella_doriae
---GCCCA
GTGAAC--TTTT-TAACGGCCGCGG-TATTCTAACCGTGCAAAGGTAGCGTAATCA
CTTGTCTTCTAAATAAAGACCAGTATGAA-CGGCTAAATGAGGGTGAACCTGTCTCCTAC
GCCCTATCAGTGAAACTGATCTTTCAGTCCAAAAGCTGAAATACCCACACAAGACGAGAA
GACCCTGTGGAGCTTAAAACCCCA-TACTAAT--AA-ACTAGTATGCCTTTTAAGTTGGG
GCGACTTCGGAACAAAACAAAACTTCCGAGCACAGA-ACC-ACC-AC-TTCT-TC-
CTAAGGCCCACACGCC-AAA--GAATTGACCCAGT-CACACT--GATCAACGAACC
AAGTTACCCCAGGGATAACAGCGCTATCTTCTTCAAGAGTCCATATCGACAAGAAGGTTT
ACGACCTCGATGTTGGATCAGGACACCCAAATGGTGCAGCCGCTATTAAAGGTTCGTTTG
TTCAACGATTAACAGTCCTACGTGATCTG

>NAP-6376_Scincella_rupicola
---GCCCA
GTGAAC--TTTTTTAACGGCCGCGG-TATTCTAACCGTGCAAAGGTAGCGTAATCA
CTTGTCTTCTAAATAAAGACCTGTATGAA-CGGCTAAATGAGGGCGGGCCTGTCTCCTGC
GCCCTATCAGTGAAACTGATCCCCCAGTCCAAAAGCTGGAATCTCCTCACAAGACGAGAA
GACCCTGTGGAGCTTTAAACCTAA-TACTAAT--GA-ACTAGTACCTGTTTTGAGTTGGG
GCGACTTCGGAACAAAATAAAACTTCCGAGCACAGA-ACC-ACT-AC-TTCT-TAG
CCGAGGCCAACAAGCC-AAA--GAACTGACCCAGT-CACACT--GATCAACGAACC
AAGTTACCCCAGGGATAACAGCGCCATCTTCTTCAAGAGTTCATATCGACAAGAAGGTTT
ACGACCTCGATGTTGGATCAGGACACCCAAATGGTGCAGCCGCTATTAAAGGTTCGTTTG
TTCAACGATTAACAGTCCTACGTGATCTG

>BAM-36_Ablepharus_deserti
AAACCCATCCCATTATCAAAATTGTAAACAGCTCATTCATCGACCTGCCATCCCCCTCTA
ACATTTCTGCCTGATGAAACTTCGGCTCACTACTAGGCTTATGCTTGATTTCCCAAACTT
TAACAGGCCTATTTTTAGCCATGCATTATACTGCAGACATCACCTCGGCCTTCTCCTCCA
TCGCCCACATCTGCCGAGACGTACAATACGGTTGACTTATCCGAAATCTTCATGCAAACG
GTGCCTCCATATTCTTTATTTGCCTCTACCTTCACATCGGCCGAGGTCTTTATTATGGCT
CTTACATATATAAAGAGACCTGAAACATCGGCGTAGTATTATTACTACTAGTAATAGCAA
CTGCCTTCGTCGGCTATGTTCTTCCATGAGGACAAATATCATTCTG

>BAM-1111_Ablepharus_kitaebelii
AAACTCACCCAATTATTAAAATTGTAAACGACTCATTCATTGACTTACCATCCCCATCAA
ACATTTCTGCTTGATGAAACTTCGGCTCTCTACTCGGACTATGCCTAATTACCCAAACCC
TCACAGGCTTATTCTTGGCCATACATTATACAGCCGATATCTCCTCCGCCTTCTCATCTA
TCGCCCATATCTGCCGAGATGTACAATATGGCTGACTTATCCGAAACCTACATGCAAACG
GGGCCTCCATATTTTTTATCTGCCTCTACCTTCATATCGGCCGAGGACTTTACTATGGTT
CATACATATATAAAGAGACCTGAAACATTGGTGTAATCCTACTTCTATTAGTTATGGCAA
CAGCCTTCGTCGGCTATGTTCTACCATGAGGACAAATATCATTCTG

>R-14554_Ablepharus_pannonicus
AGACCCACCCAATCATTAAAATCGTAAATAACTCATTCATCGACCTTCCATCCCCGTCAA
ATATTTCTGCTTGATGAAACTTTGGCTCCCTGCTTGGCCTTTGCCTTATTATACAAACCC
TCACAGGCCTATTTTTAGCCATACACTACACAGCAGACATCTCATCCGCCTTCTCCTCCA
TCGCCCATATTTGCCGTGATGTTCAATACGGGTGACTTATCCGAAATCTCCATGCAAATG
GCGCATCTATATTTTTTATTTGCCTGTACCTCCACATTGGCCGGGGGCTCTACTACGGCT
CATATATATATAAAGAAACATGGAACATCGGCGTAATCCTATTACTCCTGGTAATAGCAA
CGGCTTTCGTCGGCTATGTATTACCCTGAGGACAAATATCATTCTG

>BAM-15_Asymblepharus_eremchenkoi
AAACCCATCCAATCATTAAAATTGTAAACGACTCATTTATTGACCTACCATCCCCATCAA
ATATTTCTGCTTGATGAAACTTTGGCTCACTATTAGGCCTATGTCTAATTATACAAACTA
TTACAGGCTTATTTCTAGCCATGCACTATACAGCAGACATTTCCTCCGCCTTTTCCTCCA
TCGCCCACATCTGCCGAGATGTTCAATACGGATGACTTATACGAAACCTCCACGCAAACG
GCGCATCCATGTTCTTTATCTGCTTATATCTTCACCTGGGCCGCGGGCTCTATTATGGCT
CATATATATACAAAGAGACCTGAAACATTGGTGTAATCCTACTTCTACTAGTTATAGCAA
CAGCTTTCGTCGGTTATGTTCTACCTTGAGGACAAATATCATTCTG

>BAM-135_Asymblepharus_alaicus
AAACCCACCCCATCATTAAAATTGTAAACAATTCATTCATTGACCTACCATCCCCATCAA
ATATTTCTGCTTGATGGAACTTTGGCTCCCTACTAGGCTTATGCCTAATTATACAAACCA
TTACAGGCCTATTTCTAGCCATACACTATACAGCAGACATTTCCTCCGCCTTTTCCTCCA
TCGCCCACATCTGCCGAGATGTTCAATATGGATGACTTATACGAAACCTCCACGCAAACG
GCGCATCAATATTCTTTATCTGTTTATATCTTCACCTCGGCCGCGGGCTCTATTATGGCT

CTTATATATATAAAGAAACCTGAAACATTGGCGTAGTCCTGCTTCTATTAGTTATAGCAA
CAGCATTTGTTGGCTATGTCCTACCTTGAGGACAAATATCATTCTG

>NAP-9807_Asymblepharus_mahabharatus
AAACCCACCCAATCTTAAAAATTGTAAACGACTCATTTATTGACCTACCATCCCCATCAA
ACATTTCTGCCTGATGAAACTTTGGCTCCCTTCTGGGCCTCTGCTTAATTGCACAAACCC
TAACCGGCCTATTTTTAGCCATACACTATACAGCAGACATCTCGTCAGCCTTTGCCTCAA
TCACCCATATTTGCCGCGATGTACAATACGGTTGACTTATTCGAAACCTACACGCAAATG
GGGCCTCTATATTCTTCATTTGTTTATACCTTCACATTGGCCGAGGACTTTACTATGGCT
CCTACATATACAAAGAAACCTGAAACATTGGCGTACTTTTACTTTTACTCGTCATAGCAA
CAGCCTTTGTAGGTTACGTATTACCTTGAGGACAAATATCATTCTG

>NAP-9870_Asymblepharus_nepalensis
AAACCCACCCTGTATTAAAAATTGTAAATAACTCATTCATTGACCTACCGTCCCCATCAA
ATATTTCTGCTTGATGAAACTTTGGCTCACTACTTGGCCTATGCCTTATTGCTCAAACCA
TCACAGGCCTCTTCCTAGCCATACACTACACGGCTGACGTCTCATCCGCCTTCTCCTCCA
TCGCCCACATCTGTCGCGATGTACAATACGGTTGACTTATTCGAAACCTCCATGCAAACG
GCGCCTCTATATTTTTTATTTGCCTTTACCTTCACATCGGCCGAGGACTCTACTACGGCT
CCTACACATATAAAGAAACCTGAAATATTGGCGTCATCCTTTTACTACTCGTTATAGCAA
CAGCCTTTGTTGGCTATGTACTGCCATGAGGACAAATATCATTCTG

>NAP-9840_Asymblepharus_sikkimensis_2
AAACCCACCCCCTCATCAAAATTGTAAATGATTCGTTCATCGACCTGCCATCCCCATCAA
ATATTTCCGCCTGATGAAACTTTGGCTCACTCCTAGGCCTCTGCTTAATTGCACAAACCC
TTACCGGTTTATTCCTAGCTATGCACTATACAGCAGATATCTCCTCCGCCTTCTCATCAA
TCGCCCATATCTCCCGAGATGTACAATACGGCTGACTTATCCGGAATCTTCACGCCAACG
GCGCCTCTATATTCTTCATTTGCCTGTACCTTCACATCGGCCGAGGACTTTATTACGGCT
CATATATATATAAAGAGACCTGAAACATCGGTGTAATTCTTCTCCTACTAGTAATAGCAA
CAGCCTTTGTAGGATATGTCCTACCATGAGGACAAATATCATTCTG

>NAP-9816_Asymblepharus_sikkimensis_3
AAACCCACCCCCTCATCAAAATTGTAAACGACTCGTTCATCGACCTGCCATCTCCATCAA
ATATTTCCGCCTGATGAAACTTTGGTTCACTCCTAGGCCTCTGCTTAATTGCACAAACCC
TTACCGGCTTATTTCTAGCTATACACTATACAGCAGACATCTCCTCAGCCTTCTCATCAA
TCGCCCATATTTCTCGAGATGTTCAATACGGCTGACTCATCCGAAATCTTCATGCCAACG
GCGCCTCTATATTCTTCATTTGCCTATACCTTCACATTGGCCGAGGACTTTATTATGGCT
CATACATGTATAAAGAAACCTGAAACATCGGTGTAATTCTTCTCCTACTAGTTATAGCAA
CAGCCTTTGTAGGATATGTCCTACCATGAGGACAAATATCATTCTG

>NAP-9858_Asymblepharus_sikkimensis_1
AAACCCACCCCCTCATCAAAATTGTAAATGATTCATTCATCGACCTGCCATCCCCATCAA
ATATTTCCGCCTGATGAAACTTTGGCTCACTCCTAGGCCTCTGCTTAATTGCACAAACCC
TTACCGGTTTATTCCTAGCTATGCATTATACAGCAGACATCTCCTCCGCCTTCTCATCAA
TCGCCCATATCTCCCGAGATGTACAATACGGCTGATTAATCCGGAATCTTCACGCCAACG
GCGCCTCTATATTCTTCATTTGCCTGTACCTTCACATCGGCCGAGGACTTTATTATGGCT
CATATATATACAAAGAGACCTGAAACATCGGTGTAATTCTTCTTCTATTAGTAATAGCAA
CAGCCTTTGTAGGATATGTCCTACCATGAGGACAAATATCATTCTG

>ZM146_Protoblepharus_sp.nov.
-CCATCCA
ACATCTCAGCATGATGAAACTTCGGGTCCCTACTAGGCCTTTGTCTAATTATACAAACTC
TAACAGGCCTATTCTTAGCAATACACTACACAGCAGATATTACATCAGCCTTCTCTTCAA
TCGCCCACATCTGTCGAGACGTCCAATACGGCTGACTCATCCGAAACCTTCACGCAAACG
GCGCTTCACTCTTCTTCATTTGCCTTTACCTACATGTAGGACGTGGACTATATTACGGCT

CTTACACATACAAAGAAACCTGAAACATTGGAGTAATCCTCCTCCTACTAGTAATAGCCA
CAGCCTTCGTAGGCTATGTTCTACCATGAGGACAAATATCATTCTG

>ZM145_Protoblepharus_sp.nov.
-CCATCCA
ACATCTCAGCATGATGAAACTTCGGGTCCCTACTAGGCCTTTGTCTAATTATACAAACTC
TAACAGGCCTATTCTTAGCAATACACTACACAGCAGATATTACATCAGCCTTCTCTTCAA
TCGCCCACATCTGTCGAGACGTCCAATACGGCTGACTCATCCGAAACCTTCACGCAAACG
GCGCTTCACTCTTCTTCATTTGCCTTTACCTACATGTAGGACGTGGACTATATTACGGCT
CTTACACATACAAAGAAACCTGAAACATTGGAGTAGTCCTCCTCCTACTAGTAATAGCCA
CAGCCTTCGTAGGCTATGTTCTACCATGAGGACAAATATCATTCTG

>NAP-6640_Scincella_melanosticta
AAACCCACCCTGTTCTAAAAATCGTAAACAACTCATTCATTGACCTCCCCTCCCCTTCCA
ACATCTCAGCCTGATGAAACTTCGGATCTCTCCTCGGAGTCTGCCTAATTATACAAGTAT
TAACAGGACTATTCCTAGCCATACACTACACAGCAGACATCTCCTCAGCCTTCTCATCGA
TCGCCCACATCTGCCGTGACGTCCAATATGGTTGACTAATCCGAAACCTACATGCTAACG
GGGCCTCTATATTCTTTATTTGCATCTACCTCCACATCGGACGCGGGCTTTATTACGGCT
CATACATATATAAAGAAACCTGAAATATCGGAGTAGTGCTTCTACTTCTTGTAATAGCAA
CCGCCTTCGTAGGCTATGTCCTTCCATGAGGACAAATATCATTCTG

>NAP-1331_Scincella_doriae
AAACACACCCAATCCTAAAAATTGTCAATGATTCCTTCATTGACCTCCCCTCACCCTCAA
ACATTTCAGCCTGATGAAATTTCGGCTCCCTTTTAGGGCTCTGCCTAATTATTCAAGTTC
TTACAGGGCTCTTCTTAGCCATACACTACACCGCTGATATCTCCTCCGCCTTCTCCTCAA
TCGCCCACATCTGCCGCGATGTTCAATACGGCTGACTAATTCGAAACCTCCATGCTAACG
GCGCCTCAATATTCTTTATTTGCCTATATCTTCATATTGGACGCGGCCTATATTACGGCT
CCTACATATATAAAGAAACATGAAACATTGGCGTTATTCTCCTACTATTGGTAATAGCAA
CTGCCTTCGTCGGTTATGTCCTCCCATGAGGACAAATATCATTCTG

>NAP-6376_Scincella_rupicola
AAACCCACCCCCTCCTAAAAATTATTAATAATTCTTTTATTGACCTCCCCTCACCCTCAA
ACATCTCCGCCTGATGAAACTTCGGATCGCTTTTAGGACTTTGCCTAATTGTGCAGGTCC
TCACCGGACTATTCTTAGCCATACACTATACTGCTGATATCTCCTCAGCCTTCTCTTCAA
TTGCTCATATCTGCCGAGATGTCCAATACGGGTGGTTTATGCGAAACCTCCATGCAAACG
GCGCCTCCATATTCTTTATTTGCATTTACATTCATGTTGGACGGGGCTTATACTACGGCT
CTTATATGTACAAAGAGACATGAAACATCGGAGTAATCCTCCTCTTACTAGTAATAGCAA
CAGCCTTCGTAGGATATGTTCTCCCCTGAGGACAAATATCATTCTG

>BAM-15_Asymblepharus_eremchenkoi
CAAGAACCGGAACCTGCACTCCCCGATGTACTACTTCATCTGCTGCCTGGCTGTCTCGGA
CACGCTGGTGAGCGTCAGCAACCTGGTGGAGACCCTCTTCATGCTGCTGATCGAGCACGG
CGTCCTGGTGGCCGAGTACAACACCCTGAAGCACATGGACAACGTCATGGACATGCTGAT
CTGCAGCT--CACTGCTGTCCTCCCTCTCCTTCCTGGGGGTCATTGCCGTCGACCG
CTACATCACCATCTTCTATGCCCTGCGCTACCACAGCATCATGACCCTCCAGCGGGCGGT
GCTCATCATTGTGGTGGTCTGGCTGGCCAGCAGCATCTCCAGCACCATCTTCATCGCCTA
TGACAGCGACGCGGTCATCCTGTGCGTGGTGATCTTCTTCCTCTCCATGGTCACCCTCAT
TGTGGCGCTCTACATCCACATGTTCACCCTGGCCCACCAGCACGCCCGGCGGATCTCCAG
CCAGCAGAGGAAGCAGACCGCCCCCCACTTCACCAGCATGAAAGGGGCCGTCACCCTCAC
CATCTTGCTGGGGGTCTTCTTCGTCTGCTGGGGGCCCTTCTTCCTGCACCTGATCCTCAT
CCTCACCTGCCCCAGGCACCCGGCCTGCAGCTGCTACTTCAGCTACTTCAACCTCTACCT
CATTCTCGTCATTTGCAACTCTGTGGTGGACCCCATCATTTTCCTTT

>BAM-135_Asymblepharus_alaicus

CAAGAACCGGAACCTGCACTCCCCAATGTACTACTTCATCTGTTGCCTGGCTGTCTCGGA
CACGCTGGTGAGCGTCAGCAACCTGGTGGAGACCCTCTTCATGCTGCTGATCGAGCACGG
CGTCCTGGTGGCCGAGTACAACACCCTGAAGCACATGGACAACGTCATGGACATGCTGAT
CTGCAGCT--CCCTGCTGTCCTCCCTCTCCTTCCTGGGGGTCATTGCCGTCGACCG
CTACATCACCATCTTCTATGCCCTGCGCTATCACAGCATCATGACCCTCCAGCGGGCGGT
GCTCATCATTGTGGTGGTCTGGCTGGCCAGCAGCATCTCCAGCACCATTTTCATCGCCTA
CGACAGCGACGCGGTCATCCTGTGCGTGGTGATCTTCTTCCTCTCCACGGTCACCCTCAT
TGTGGCGCTCTACATCCACATGTTCACCCTGGCCCACCAGCACGCCCGGCGGATCTCCAG
CCAGCAGAGGAAGCAGACCGCCCCCCACTTCACCAGCATGAAAGGGGCCGTCACCCTCAC
CATCTTGCTGGGGGTCTTCTTCGTCTGCTGGGGGCCCTTCTTCCTGCACCTGATCCTCAT
CCTCACCTGCCCCAGGCACCCGGCCTGCAGCTGCTACTTCAGCTACTTCAACCTCTACCT
CATTCTCGTCATTTGCAACTCTGTGGTGGACCCCATCATTTACCCTT

>BAM-36_Ablepharus_deserti
CAAGAACCGGAACCTGCACTCCCCGATGTACTACTTCATCTGCTGCCTGGCTGTCTCGGA
CACGCTGGTGAGCGTCAGCAATCTGGTGGAGACCCTCTTCATGCTGCTGATCGAGCACGG
CGTCCTGGTGGCCGAGTACAACACCCTGAAGCACATGGACAACGTCATGGACATGCTGAT
CTGCAGCT--CCCTGCTGTCATCCCTCTCCTTCCTGGGGGTCATTGCCGTCGACCG
CTACATCACCATCTTCTATGCCCTGCGCTACCACAGCATCATGACCCTCCAGCGGGCGGT
GCTCATCATTGTGATGGTCTGGCTGGCCAGCAGCATCTCCAGCACCATCTTCATCGCCTA
CGACAGCGACGCGGTCATCCTGTGCGTGGTGATCTTCTTCCTCTCCATGGTCACCCTGAT
TGTGGCGCTCTACATCCACATGTTCACCCTGGCCCACCAACACGCCCGGCGGATCTCCAG
CCAGCAGAGGAAGCAGACCGCCCCCCACTTCACCAGCATGAAAGGGGCCGTCACCCTCAC
CATCTTGCTGGGGGTCTTCTTCGTCTGCTGGGGGCCCTTCTTCCTGCACCTGATCCTCAT
CCTCACCTGCCCCAGGCACCCGGCCTGCAGCTGCTACTTCAGCTACTTCAACCTCTACCT
CATTCTCGTCATTTGCAACTCTGTGGTGGACCCCATCATTTACGCTT

>NAP-9870_Asymblepharus_nepalensis
CAAGAACCGGAACCTGCACTCCCCAATGTACTACTTCATCTGCTGCCTGGCTGTCTCAGA
CACGCTGGTGAGCGTCAGCAACCTGGTGGAGACCCTCTTCATGCTGCTGATCGAGCATGG
CGTCCTGGTGGCCGAGTACAACACCCTGAAGCACATGGACAACGTCATGGACATGCTGAT
TTGCAGCT--CCCTGCTGTCGTCCCTCTCCTTCCTGGGGGTCATTGCCGTTGACCG
CTATATCACCATCTTCTATGCCCTGCGCTACCACAGCATCATGACCCTCCAGCGGGCGGT
GCTCATCATCGTGATGGTCTGGCTGGCCAGCAGCATCTCCAGCACCATCTTCATCGCCTA
CGACAGCGACGCGGTCATCCTGTGCGTGGTGATCTTCTTCCTCTCCATGGTCACCCTCAT
CGTGGCACTCTACATCCACATGTTCACCCTGGCCCACCAGCATGCCCGGCGGATCTCCAG
CCAGCAGAGGAAGCAGACCGCCCCCCACTTCACCAGCATGAAAGGGGCCGTCACCCTCAC
CATCTTGCTGGGGGTCTTCTTCGTCTGCTGGGGGCCCTTCTTCCTGCACCTGATCCTCAT
CCTCACCTGCCCCAGGCACCCGGCCTGCAGCTGCTACTTCAGCTACTTCAACCTCTACCT
CATTCTTGTCATTTGTAACTCTGTGGTGGACCCCATCATTTACGCTT

>NAP-9807_Asymblepharus_mahabharatus
CAAGAACCGGAACCTGCACTCCCCAATGTACTACTTCATCTGCTGCCTGGCTGTCTCAGA
CACGCTGGTGAGTGTCAGCAACCTGGTGGAGACCCTCTTCATGCTGCTGATCGAGCACGG
CGTCCTGGTGGCCGAGTACAACACCCTGAAGCACATGGACAACGTCATGGACATGCTGAT
CTGCAGCT--CCCTGCTCTCGTCCCTCTCCTTCCTGGGGGTCATTGCCGTCGACCG
CTACATCACCATCTTCTACGCCCTGCGCTACCACAGCATCATGACCCTCCAGCGGGCGGT
GCTCATCATTGTGGTGGTCTGGCTGGCCAGCAGCATCTCCAGCACCATCTTCATCGCCTA
CGATAGTGACGCGGTCATCCTGTGCGTGGTGATCTTCTTCCTCTCCATGGTCACCCTCAT
CGTGGCGCTCTACATCCACATGTTCACCCTGGCCCACCAGCACGCCCGGCGGATCTCCAG
CCAGCAGAGGAAGCAGACCGCCCCCCACTTCACCAGCATGAAAGGGGCCGTCACCCTCAC
TATCTTGCTGGGGGTCTTCTTCGTCTGCTGGGGGCCCTTCTTCCTGCACCTCATCCTCAT
CCTCACCTGCCCCCGGCACCCGGCCTGCAGCTGCTACTTCAGCTACTTCAACCTCTACCT
CATCCTTGTCATTTGCAACTCTGTGGTGGACCCCATCATTTTCCCTT

>NC054206_Scincella_reevesii
---CTGCTGTGTGGCCGGGTCGTA
TTCGGAGGTGAGCGTGAGCGTCATGGCCTAGACGGTCTTCATGTTGCTGATGCTGATCGG
GCTGCTGGTGCTGGTGGCCGACTACCTCACGCTGAAGCACATGGTCAAGGACATGCACAT
GCGCATCT--CCCTGATGTCGACCCCCTCCTTCCCGGGCGTGGTCGTCATCGACCG
CTACATCAACATCTTCTACTTCCAGGCCTAGCACAACAACATGACCCTGACCCTGGCGGT
GGTCATGATGATCATGGTCTGGGTGGGGATGGCCATCACCATCACCCTCTTCATCTTCAA
CGACAACGACACCGACATCCTGTGCCTGGTCATGTTCATCCTCTTCATGGGCACGGTCAT
CCTGGTCCTGGACCTGCACATGTTCATGTTGGCCCAGCAGCACCCCCACCGGATCTCGAT
CCTGCACCTGAAGCCGAACCCCACCCCCTTCCACTTCCTGAACGGGACCGTCGCCCTCAC
CCTGCTGCTGGGGGTGGTGGTCTTCTGCTGGGGGCGGGTGTTCTTCCTCCTCAACCTCAT
CCTCATCTGCACCAGGCACACGCACTGCACCTGCTACTTCAACTACTTCTACTTCAACCT
CAACCTGATCATGGTCAACTGCAAGGTGGAGCTGAACACTAACATCT

>NHMC80.3.131.57_Ablepharus_anatolicus
CTCTACTAATGAACCCTATTTTAAATTCTTTGATTATCTCAAACCTCGCCCTAGGNACAAACAT
TACACTATCTAGCTACCATTGATTATTCGCCTGGCTAGGCCTAGAACTTAATACATTAGC
GATTATTCCAATTATTGCACAACAACATCATCCCCGAGCAACCGAAGCAGCAACAAAATA
TTTCCTCATTCAAGCAGCCGCCTCAGCAATACTACTATTTGCCAGTACCCTAAACGCCTG
ATCAACAGGCACCTGAAATATTACAGAACTCTCAAATCAATCCGCCTCCATTATATTAAC
CATGGCCCTCGCAATAAAACTAGGACTTGCCCCATTACACTTCTGATTACCAGAAGTCCT
ACAAGGTTCTACTACAATAACCGCACTAATTATCGCCACATGACAAAAACTAGCTCCCCT
TGCTCTATTATATCTTACATATAACACATTACACCCCACAACTTTACTAATCATAGCTTT
AATGTCCACCCTTGTGGGCGGTTGAGGCGGTCTAAACCAAACCCAACTA---

>BAM-36_Ablepharus_deserti

CTCTACTAATGAATCCAGTAATAACCTCATTAATTATCTCAAGCCTAGCCCTAGGCACGATCAT
CACAATGTCTAGCCACCATTGACTACTCGCCTGACTGGGCCTAGAACTTAATACCCTAGC
AATTATTCCAATCATCGCACACCAACATCACCCACGAGCAACTGAAGCCGCAACAAAATA
TTTCCTGACCCAAGCAGCTGCATCAGCCATACTCTTATTTGCCAGCACCACAAACGCCTG
ATCCACTGGCACCTGAAATATTCTAGAACTATCAAACCTACCCGCCACCATTATACTAAC
AACAGCCCTAGCAATAAAACTAGGCCTAGCCCCACTACACTTCTGATTACCTGAAGTCCT
ACAGGGCTCAACTATCAAGACCGCACTAATTCTTACCACCTGACAAAAGTTAGCCCCAAT
GACCATCCTTTACCTAACATACAATTCACTTCATCCTATAACACTACTAATCATAGCCCT
TATATCAAGCCTTATTGGTGGCTGAGGTGGCCTAAACCAAACCCAACTA---

>BAM-1111_Ablepharus_kitaebelii
CTCTTCTAATGAACCCTGTTATAAATTCCTTAATCATTTCAAGCCTTGCCCTAGGNACTATAAT
TACACTGTCCAGCCACCACTGATTACTTGCTTGACTTGGCCTTGAACTAAATACCCTAGC
AATTATCCCAATTATCGCACATCAACATCACCCCCGCGCAACCGAAGCTGCAACAAAGTA
CTTTCTTATTCAAGCAGCTGCCTCAGCCATACTACTATTTGCCAGCACCACAAATGCCTG
ATCCACAGGCACCTGAAACATTATAGAACTCTCAAACCAACCCGCTTCCATTATATTAAC
CATAGCTCTAACAATAAAATTAGGACTTGCCCCATTACACTTCTGACTACCAGAAATTCT
ACAGGGCTCCACTACCAAAACTGCATTGATTATCGCCACATGACAAAAATTAGCCCCCAT
AACCCTACTATACCTTACATACAACTCACTACACTCAACAACACTACTAATTATAGCCCT
AATATCAACCCTTGTCGGCGGCTGAGGTGGCCTCAACCAAACCCAACTA---

>NHMC80.3.82.2_Ablepharus_kitaibelii

CTCTACTAATGAACTCCCTTATAAACTCCTTAATCATTTCAAGCCTTGCCCTTGGNACAAAAAT
TACACTGTCCAGCCACCACTGATTACTTGCTTGACTTGGCCTAGAATTAAATACCCTAGC
AATTATCCCAGTTATCGCACACCAACATCACCCCCGCGCAACCGAAGCAGCAACAAAGTA

CTTTCTCATCCAAGCAGCTGCCTCAGCCATGTTACTATTTGCCAGCACCACAAATGCCTG
ATCCACGGGCACCTGAAATATTATAGAACTCTCAAACCAATCCGCTTCCATTATATTAAC
CATGGCTCTGACAATAAAATTAGGACTTGCCCCATTACACTTCTGATTACCAGAAATTTT
ACAAGGCTCCACCACCAAAACTGCATTAATTATCGCTACATGACAAAAATTAGCCTCTCT
AACCCTACTATACCTTACATATAATTCATTACACCCAACAACACTTCTAATTATAGCCTT
AATATCAACCCTTGTCGGCGGCTGAGGTGGCCTCAACCAAACCCAACTA---

>R-14554_Ablepharus_pannonicus

CTCTACTAATGAACCCAATTATAACTTCACTAATTATCTCAAGCCTCGCCTTAGGTACTATTAT
TACACTAACTAGTTATCACTGATTATTAGCCTGACTTGGCCTAGAACTTAATACCCTAGC
AATTATTCCGATTATTACACAACAACACCACCCACGAGCAACAGAAGCAGCAACAAAATA
CTTCCTAACTCAAGCTGCAGCCTCAGCTATACTAATATTTGCTAGCACCACGAACGCCTG
AACTACTGGCAGCTGAAACATTCTAGAACTCTCCAGCCAACCAGCCTCTATTATATTAAC
CATAGCCTTAGCAATAAAACTAGGACTCGCCCCACTTCACTTCTGATTGCCTGAAGTCCT
ACAAGGTTCCACCATCAAAACAGCATTAATTATTACAACATGACAAAAACTCGCCCCACT
AGCTCTTCTTTACCTAACACACAACATGCTACACCCCACAATATTATTAATTATAGCCCT
AATATCAAACCTCATTGGCGGTTGAGGCGGATTAAACCAAACCCAACTA---

>NHMC80.3.169.5_Ablepharus_rueppellii
CTCTACTAATGAATCCCAGTTTTAACTCCTTACTTATCTCAAACCTCGCCCTCGGTACTATTAT
TACTCTATCTAGCCACCACTGACTACTCGCCTGACTAGGATTAGAACTAAACACCCTAGC
AATTATCCCCATTATTGCACACCAACATCACCCACGCGCAACCGAAGCGGCAACAAAATA
TTTCCTCACTCAGGCAGCCGCTTCGGCTATACTTCTATTTGCCAGCACCATTAATGCATG
GTCTATGGGTACATGAAACATCTTAGAGCTCTCAAGTCAACCCGCTTCCATTATATTAAC
AATAGCCCTGGCAATAAAACTAGGACTTGCCCCTCTACACTTCTGATTGCCGGAGGTCTT
ACAAGGCTCTACAACCATAACAGCACTAATTATCACCACTTGACAAAAACTAGCCCCCCT
CGCCCTTCTCTACCTTACATATAACACACTGCACCCCCCAACACTACTAACTATGGCCCT
AATATCAACACTTGTCGGCGGCTGAGGGGGCCTCAACCAAACCCAACTA---

>BAM-15_Asymblepharus_eremchenkoi
CTCTACTAATGAATCCAATTATAGCCTCACTAATTATATCAAGCCTAGCCATAGGCACAATCAT
CACACTATCTAGCTACCACTGATTATTAGCCTGACTGGGCCTAGAACTTAATGCTTTAGC
AATTATTCCCATTATTGCTAAACAACACCACCCCCGCGCAACAGAAGCAGCAACAAAATA
TTTCCTTACTCAGGCAGCCGCATCAGCATTACTCCTATTCGCAAGTATTATTAATGCTTG
AGCCACCGGAACCTGGAACATTATTGAATTTTCTGACCCGCAAGCCTCTATCATACTAAC
TACCGCCCTAGCATTAAAATTAGGCCTTGCCCCACTACACTTCTGATTACCAGAGGTTTT
ACAAGGCATCACTATAAAAACTGCACTAATTATTACCACATGACAAAAACTAGCCCCACT
AACCCTTTTTTACTTAACATATAACTCACTACACCCCATAACACTACTGATTATAGCCCT
TCTATCAAACTTCATCGGCGGTTGAGGCGGCTTAAACCAAACCCAACAA---

>BAM-135_Asymblepharus_alaicus

CTCTACTAATGAATCCNATTATAACCTCACTAATTATCTCAAGCCTAGCCATAGGTACAATCAT
CACACTATCGAGCTACCACTGATTATTGGCCTGAATGGGCCTAGAACTAAATGCCTTAGC
AGTTATTCCCATTATTGCCAAACACCACCACCCCCGCGCAACAGAAGCAGCAACAAAATA
CTTCCTTACCCAAGCAGCCGCATCAGCATTACTCCTATTCGCAAGTATTATCAATGCTTG
ATCCACCGGAACCTGAAGTATTATTGAACTTTCTAACCCACAAGCCTCTATTATATTAAC
GACTGCCCTCGCATTAAAATCAGGCCTTGCCCCACTACACTTCTGATTGCCAGAAGTTTT
ACAAGGCACCACCATACAAACTGCACTAATTATTGCCACATGACAAAAACTAGCCCCACT
AACCCTTTTTTATTTAACATATGACTCACTACACCCCATAACGCTACTGATTATAGCCCT
ACTATCAAACTTCATTGGCGGCTGAGGCGGCTTAAACCAAACCCAACTA---

>NAP-9807_Asymblepharus_mahabharatus

CTTTACTAATGAACCCTTTAATAACCTCTTTAATTATCTCAAGTTTAGCCCTAGGCACAATCAT
TACACTATCTAGCCACCATTGATTGTTAGCCTGACTAGGCTTAGAACTTAATACACTCGC
AATTATTCCCCTAATCGCAGAACAACATCATCCACGTGCAACCGAAGCAGCTACAAAGTA
TTTCCTTACACAAGCAGCCGCATCAGCACTACTTCTATTTGCCAGCACCACAAACGCCTG
ACTTACAGGCACCTGAAATATTATCGAATTATCAAGTCAACCAGCATCCATTATATTAAC
TATAGCACTGGCAATAAAATTAGGCCTTGCCCCACTCCACTTCTGATTACCAGAGGTATT
ACAAGGCACCACCATGAAAACAGCACTAATCATTACCACATGACAAAAACTAGCCCCATT
AGCCCTATTATACTTAACATACAATACTTTACACCCTATAACACTATTAACAATAGCCCT
TCTATCAAACATCCTCGGTGGGTGAGGCGGCCTGAACCAAACCCAACAA-

>NAP-9870_Asymblepharus_nepalensis
CTTTACTAATGAACCCCATAATAATAACCCTTATTATTTCTAGCCTTGCAACAGGCACAATCAT
CACACTATCAAGCTACCATTGATTACTCGCCTGAATCGGACTAGAACTTAATGCTCTATC
AATCATTCCAATCATCGCAAAACACCACCACCCCCGCGCAACCGAAGCAGCAACAAAATA
CTTCCTCACACAAGCAGCTGCATCAGCACTATTATTATTTCAAGCACTACAAATGCTTG
AATTACAGGCACCTGAAATATTACTGATATCTCAAATCAACCAGCCTCAATTATACTAAC
AATAGCCCTTGCAATAAAATTAGGACTTGCTCCCATACACTTCTGACTCCCAGAAGTACT
ACAAGGCACCACCATTAAAACAGCATTAATTATTACCACATGACAAAAATTGGCCCCATT
AGCCCTCTTCTACTTAACACATAATGCACTACACCCAACAACACTACTAACTATAGCCAT
CCTATCAAACATCATCGGCGGCTGAGGTGGCCTCAACCAAACCCAACAA

>NAP-9840_Asymblepharus_sikkimensis_2
CTTTACTAATGAACCCTATAATAACCTCTTTAATTATCTCCAGCCTTGCCCTAGGTACAATTAT
TACACTATCTAGTTACCATTGATTATTAGCCTGAGTTGGCCTAGAACTCAATACCCTAGC
AATTATTCCCATTATTGCACAACAACATCACCCACGCGCAACCGAAGCAGCAACTAAATA
TTTCCTCACCCAAGCAGCCGCATCAGCACTAGTACTATTTGCAAGTACTACAAATGCCTG
ATTTACTGGCACCTGAAATATCACTGAACTATCGAACCAGCCGGCCTCCATCATATTAAC
TATAGCCTTAGCAATAAAACTAGGACTTGCCCCACTACACTTCTGACTCCCAGAGGTATT
ACAAGGCACTACTATAAAAACAGCACTGATTATTACCACATGACAAAAACTGGCCCCCAT
CACCCTCCTTTACCTTACACATAATTCACTCCACCCAACAACGCTTCTACTCTTGGCCCT
AACATCAAACATTATCGGCGGTTGAGGGGGACTAAACCAAACCCAACTA

>NAP-9816_Asymblepharus_sikkimensis_3
CTTT
ACTAATGAATCCTATAATAGCCTCTTTAATCATCTCCAGCCTTGCCCTAGGCACAATCAT
CACACTATCTAGTTACCATTGATTATTAGCCTGAGTTGGCTTAGAACTAAATACCCTAGC
AATTATTCCTATTATTGCACAACAACATCACCCCCGCGCAACCGAAGCAGCAACTAAATA
TTTCCTCACCCAAGCAGCTGCATCAGCACTAGTATTATTTGCAAGTACTACAAATGCTTG
ATTTACTGGCACCTGAAATATCACTGCACTATCAAACCAACCAGCCTCCATCATATTAAC
TATAGCATTAGCAATAAAACTAGGACTTGCCCCACTACACTTCTGACTCCCAGAGGTATT
ACAGGGCACTACTATAAAAACAGCACTAATTATTATCACATGACAAAAACTGGCCCCCCT
CACACTCCTTTACCTTACATATAATTCACTGCACCCTCTAACGCTGCTACTCATAGCCCT
AACATCAAACATTATCGGTGGTTGAGGGGGCCTAAACCAAACCCAACTA-

>NAP-9858_Asymblepharus_sikkimensis_1

CTTTACTAATGAATCCTATAATAACCTCTTTAATTATCTCCAGCCTTGCCCTAGGTACAATTAT
TACACTATCTAGTTACCATTGATTATTAGCCTGAGTTGGCTTAGAACTCAATACCCTAGC
AATTATTCCCATTATTGCACAGCAACATCACCCACGCGCAACCGAAGCAGCAACTAAATA
TTTCCTCACCCAAGCAGCCGCATCAGCACTAGTACTATTTGCAAGTACTACAAATGCCTG
ATTTACTGGCACCTGAAATATCACTGAACTATCAAACCAGCCGGCCTCCATCATACTAAC
TATAGCATTAGCAATAAAACTAGGACTTGCCCCACTACACTTCTGACTCCCAGAGGTATT
ACAAGGCACTACTATAAAAACAGCACTGATTATTACCACATGACAAAAACTGGCACCCCT
CACGCTCCTTTACCTTACACATAATTCACTACACCCAACAACGCTTCTACTCTTGGCCCT

AACATCAAACATTATCGGCGGTTGGGGGGACTAAACCAAACCCAACTA---

>ZM146_Protoblepharus_sp.nov.
CTCT
ACTAATGAATCCCATTATAACCTCACTGATCTTGTCAAGCCTTGCTTTAGGCACAATTAT
CACAATATCAAGTTATCACTGACTCCTAGCCTGAATTGGACTAGAACTAAATACACTAGC
GATTATTCCTCTTATCGCAAAACAACACCACCCACGAGCAACCGAAACCGCAACGAAATA
CTTCCTCATCCAAGCTGCAGCCTCAGCAACAC

>ZM145_Protoblepharus_sp.nov.
CTCTACTAATGAATCCCATTATAACCTCACTGATCTTGTCAAGCCTTGCTTTAGGCACAATTAT
CACAATATCAAGTTATCACTGACTCCTAGCCTGAATTGGACTAGAACTAAATACACTAGC
GATTATTCCTCTTATCGCAAAACAACACCACCCACGAGCAACCGAAACCGCAACGAAATA
CTTCCTCATCCAAGCTGCAGCCTCAGCAACAC

>NAP-6640_Scincella_melanosticta

CTCTACTAATGAACCCAATTATAACCTCTTTAATTATTTCTAGCCTAGCCCTGGGCACAATGAT
TACAATATCAAGCTTCCACTGACTCCTAGCCTGAATTGGACTAGAGTTAAATACTATTGC
CATCCTACCCATCATTGCAAAACAACACCACCCCCGATCAACAGAGGCCACTACCAAATA
CTTCCTAACCCAAGCAGCCGCCTCAGCTACCGTACTATTTGCCAGCACAATCAATGCCTG
ATCTACCGGGACCTGAGACATTACACAACTTACAAACCAACCAGCCTCCATTATACTTAC
AATAGCCCTAGCAATAAAACTAGGGCTAGCCCCACTACACTTCTGACTTCCAGAAGTACT
ACAAGGCTCACCATTAAAAACAGCATTAATTATTACCACCTGACAAAAACTGGCCCCTAT
AGCCCTATTATTCCTAACCTATAACTCACTACACCCCACAACACTGCTCGCAATAGGACT
ACTATCAACCGTAACCGGAGGATGGGGCGGCCTCAACCAAACCCAACAA---

>NAP-1331_Scincella_doriae

CTCTACTAATGAACCCTATTATAATATCTCTAATCATTTCAAGCCTTGCTCTTGGAACCATCAT
CACAATGTCCAGCTTTCACTGATTACTTGCCTGAATTGGACTAGAACTTAATACCCTCGC
TATTATTCCAATTATTGCAAAACAACACCATCCCCGTGCAACAGAAGCAGCCACAAAATA
CTTCTTAATTCAAGCAGCAGCTTCAGCTACCATTTTATTTTCAAGCACCACCAACGCTTG
ATCCACGGGATCATGAGACATCCTACAAATAACAAACCAACCAGCTACTATTATACTCAC
TATAGCACTAACCATAAAACTAGGCCTCGCCCCCCTACACTTTTGATTACCAGAGGTACT
ACAAGGAACACCCTTAAAAACAGCATTAGTTATCACCACCTGACAAAAACTCGCACCTAT
GACCCTTTTTTTATTGGTTTACCCCACACTAAGCCCTACAACACTTTTATTAGTAGGACT
ACTCTCAACCGCCATTGGCGGCTGAGGCGGCCTAAACCAAACCCAACAC---

>NAP-6376_Scincella_rupicola
CTCT
ACTAATGAACCCCTTTATAATATCCCTGATTATTTCGAGCCTTGCCCTTGGCACCCTAAT
CACAATAACAAGCTTTCACTGGTTTCTCGCCTGAGTGGGACTAGAACTTAATACCCTCGC
TATTATCCCTATTATCGCAAAACAACACCACCCACGAGCAACTGAAGCCGCCACAAAATA
CTTCTTAATCCAGGCAGCAGCCTCGGCCACTGTACTTTTTTCAAGCGCCACTAATGCCTG
ACACACGGGCTCTTGGGATATTATACAACTAACAAATCAACCAGCAAGCATTATATTAAC
TATGGCCCTGGCCATAAAACTGGGCCTCGCCCCCCTACACTTCTGGCTCCCAGAGGTCTT
ACAAGGCGCCTCTTTAAAAACAGCCTTAATCATTGTTACCTGACAAAAACTAGCACCCCT
CGCCCTCTTTCTAATAGTTTACCCTACATTAAACCCGACAACCCTCTTAATAATGGGACT
CTTATCAACCCTGGTGGGGGGATGAGGGGGCCTAAACCAAACCCAACAC---

>BAM-36_Ablepharus_deserti
TCTCCAGTAAGTCTGCGGGTGAACTTCCAGACTGTTGGTAAGAGCACCGCTGCATCAAAT
AACTCTGAAGCAAGGAATGTAAAGCCAGAAGTAGATG--GGAAA

GAGAGGGAGAA--CCCCAACCCCACCGCTACAATTCCTGTTGCA
GAAAGTGCGCTAAAAACCGAAATGGCTGAAAATGCCCAGAGCAGCATGGTGGATAACAAA
TGGAAGCCGCTCCAAGGTGTCGGGAATCTCCAGGGGGCTGTTGCAGCTGCAGCGGCTGCC
AAT-CCTTTAGAAGCCAAAAACGTGGCATCCTCATCCTCCGAATCAAAACCA
CAAGGCTTAAGGATAGAGATTAAAAGCAAAAACAAAATCAGACCAGGCTCTCTGTTTGAT
GAAGTTAGAAAGACGGCAAGGCTCAATCGGCGGCCAAGAAACCGCGAGAGCTCCAGTGAG
GAGGATTCTCCTGCACGAGAGAACAGCCCGTCCAGGAGCCGCAGCCGGTCACGGGCTAAA
TCGGATCCTAAATCCAGGCACAGAACAAGATCCCTCTCCTACAGTCACTCAAG

>BAM-15_Asymblepharus_eremchenkoi
TCTCCAGTAAGTCTGAAGGTGAGCTTCCAGACTGTTGGTAAGAGCACCGCTGCATCAAAT
AACTCTGAAGCAAGGAATGCAAAGCCAGAAGTAGATGCGAAAGAATTGGCTGATGTAAAA
GAGAGCAAAAAAGAGAGGGAGAAACCAAGCCCCAACCCCACCGCTACAATTCCTGTTGCA
GAAAGTGCACTAAAAACCGAAATGGCTGAAAATGCCCAGTGCACCATGGTGGATAACAAA
TGGAAGCCGCTCCAGGGTGTCGGAAATCTCCAGGGGGCTGTTGCAGCTGCAGCAGCTGCC
AAT-CCTTTAGAAGCCAAAAACGCGGCATCCTCATCCTCCGAATCAAAACCA
CAAGGTTTAAGGATAGAAATTAAAAGCAAAAACAAAATCAGGCCAGGCTCTCTGTTTGAT
GAAGTTAGAAAGACGGCAAGGCTCAATCGGCGGCCAAGAAACCGCGAGAGCTCCAGTGAG
GAGGATTCTCCTGCACGAGAGAACAGCCCGTCCAGGAGCCGCAGCCGGTCACGGGCTAAA
TCGGATCCTAAATCTAGGCACAGAACAAGATCCCTCTCCTATAGTCACTCAAG

>BAM-135_Asymblepharus_alaicus

TCTCCAGTAAGTCTGAAGGTGAGCTTCCAGAGTGTTGGTAAGAGCACCGCTGCATCAAAT
AACTCTGAAGCAAGGAATGCAAAGCCAGAAGTAGACGCGAAAGAATTGGCTGATGTGAAA
GAGAGCAAAAAAGAGAGGGAGAAACCAAGCCCCAACCCCACCGCTACAATTCCTGTTGCA
GAAAGTGCGCTAAAAACCGAAATGGCTGAAAATGCCCAGAGCAGCATGGTGGATAACAAA
TGGAAGCCACTCCAGGGTGTTGGAAATCTCCAGGGGGCTGTTGCAGCTGCAGCAGCTGCC
AAT-CCTTTAGAAGCCAAAAACGCGGCATCCTCATCCTCCGAATCAAAACCA
CAAGGTTTAAGGATAGAAATTAAAAGCAAAAACAAAATCAGGCCAGGCTCTCTGTTTGAT
GAAGTTAGAAAGACGGCAAGGCTCAATCGGCGGCCAAGAAACCGCGAGAGCTCCAGTGAG
GAGGATTCTCCTGCACGAGAGAACAGCCCGTCCAGGAGCCGCAGCCGGTCACGGGCTAAA
TCGGATCCTAAATCTAGGCACAGAACAAGATCCCTCTCCTATAGTCACTCAAG

>NAP-9807_Asymblepharus_mahabharatus
TCCCCAGTAAGTCTGAAGGTGAACTTCCAGACTGCTGGCAAGAGCACCGCTGCATCGAAT
AACTCCGAAGCAAGGAATGCAAAGCCACAAGTAGATGCGAAAGAACTGGCTGATGTGAAA
GAGAGCAAAAGAGA---GAGGAAACCAAGCCCCAACTCCACCGCTACAATTCCTGTGGCA
GAAAGTGGGCTAAAAACTGAAATGGCTGAAAATGCCCAGAGCAGCTTGGTGGATAACAAA
TGGAAGCCGCTCCAGGGTGTCGGGAATCTCCAGGGGGCTGTTGCAGCAGCAGCTGCTGCC
AAT-CTTTTAGAAGCCAAAAACGCAGCATCCTCATCCTCCGAATCAAGACCA
CAGGGCCTAAGGATAGAAATTAAAAGCAAAAACAAAATCAGGCCAGGCTCTCTGTTTGAT
GAAGTCAGAAAGACGGCAAGGCTCAATCGGCGGCCAAGAAACCGCGAGAGCTCCAGTGAG
GAGGATTCTCCTGCCCGAGAGAACAGCCCCTCCAGTAGCCGCAGCCGGTCACGGGCGAAA
TCGGATCCCAAATCCAGACACAGAACAAGATCCCTCTCCTATAGTCACTCAAG

>NAP-9870_Asymblepharus_nepalensis
TCTCCAGTAAGTCTGAAGGTGAACTTCCAGACTGCTGGTAAGAGCACCGCTGCATCAAAT
AACTCTGAAGCAAGGAATGCAAAGCCAGAAGTAGATGGGAAAGAATTGGCTGATGGGAAA
GAGAGCAAAAAAGAGAGGGAGAAACCAAGCCCCAACCCCAGCGCTACAATTCCTGTTGCA
GAAAGTGTGCTAAAAACTGAAATGGCTGAAAATGCCCAGAGTAGCATGGTGGATAACAAA
TGGAAGCCGCTCCAGGGTGTCGGGAATCTCCAGGGGGCTGTTGCAGCTGCAGCAGCTGCC
AAT-CCTTTAGAAGCCAAAAACGCGGCATCCTCATCCTCTGAATCAAGACCA
CAAGGCTTAAGGATAGAAATTAAAAGCAAAAACAAAATCAGGCCAGGGTCTCTGTTTGAT

GAAGTTAGAAAGACGGCAAGGCTCAATCGGCGGCCAAGAAACCGCGAGAGCTCCAGTGAG
GAGGATTCTCCTGCACGAGAGAACAGCCCCTCCAGGAGCCGCAGCCGGTCACGGGCGAAA
TCGGATCCTAAATCCAGGCACAGAACAAGATCCCTCTCCTATAGTCACTCAAG

>NAP-9840_Asymblepharus_sikkimensis_2
TCTCCAGTGGGTCTGAAGGTGAACTTCCAGACTGTTGGTAAGAGCACCGCTGCATCAAAT
AACTCTGAAGCAAGGAATGCAAAGCCAGGAGTAGATGCGAAAGAATTGGCTGATGTGAAA
GAGAGCAAAAGAGAGAGG---AAACCAAGCCCCAACCCCACCGCTACAATTCCTGCTGCA
GAAAGTGTGCTAAAAACGGAAATGGCTGAAAATGCCCAGAGCAGCGTGGTGGATAACAAA
TGGAAGCCGCTCCAGGGTGTCGGGAATCTCCAGGGGGCTGTTGCAGCAGCAGCCGCTGCC
AAT-CTTTTTGAAGCCAAAAACGCAGCATCCTCATCCTCCGAATCAAGGCCA
CAAGGCTTAAGGATAGAAATTAAAAGCAAAAACAAAATCAGGCCAGGCTCTCTGTTTGAT
GAAGTCAGAAAGACGGCAAGGCTCAATCGGCGGCCCAGAAACCGCGAGAGCTCCAGTGAG
GAGGATTCTCCTGCACGAGAGAACAGCCCCTCCAGTAGCCGCAGCCGGTCACGGGCGAAA
TCGGATCCTAAATCCAGGCACAGAACAAGATCCCTCTCCTATAGTCACTCAAG

>NAP-9816_Asymblepharus_sikkimensis_3
TCTCCAGTGGGTCTGAAGGTGAACTTCCAGACTGCTGGTAAGAGCACCGCTGCATCAAAT
AACTCTGAAGCAAGGAATGCAAAGCCCGGAGTAGATGCGAAAGAATTGGCTGATGTGAAA
GAGAGCAAAAGAGA---GAGGAAACCAAGCCCCAACCCCACCGCTACAGTTCCTGCTGCA
GAAAGTGTGCTAAAAACGGAAATGGCTGAAAATGCCCAGAGCAGCGTGGTGGATAACAAA
TGGAAGCCGCTCCAGGGTGTCGGGAATCTCCAGGGGGCTGTTGCAGCAGCAGCCGCTGCC
AAT-CTTTTTGAAGCCAAAAACGCAGCATCCTCATCCTCCGAATCAAGGCCA
CAAGGCTTAAGGATAGAAATTAAAAGCAAAAACAAAATCAGGCCAGGCTCTCTGTTTGAT
GAAGTCAGAAAGACGGCAAGGCTCAATCGGCGGCCCAGAAACCGCGAGAGCTCCAGTGAG
GAGGATTCTCCTGCACGAGAGAACAGCCCCTCCAGTAGCCGCAGCCGGTCACGGGCGAAA
TCGGATCCTAAATCCAGGCACAGAACAAGATCCCTCTCCTATAGTCACTCAAG

>NAP-9858_Asymblepharus_sikkimensis_1
TCTCCAGTGGGTCTGAAGGTGAACTTCCAGACTGCTGGTAAGAGCACCGCTGCATCAAAT
AACTCTGAAGCAAGGAATGCAAAGCCAGGAGTAGATGCGAAAGAATTGGCTGATGTGAAA
GAGAGCAAAAGAGA---GAGGAAACCAAGCCCCAACCCCACCGCTACAATTCCTGCTGCA
GAAAGTGTGCTAAAAACGGAAATGGCTGAAAATGCCCAGAGCAGCGTGGTGGATAACAAA
TGGAAGCCGCTCCAGGGTGTCGGGAATCTCCAGGGGGCTGTTGCAGCAGCAGCCGCTGCC
AAT-CTTTTTGAAGCCAAAAACGCAGCATCCTCATCCTCCGAATCAAGGCCA
CAAGGCTTAAGGATAGAAATTAAAAGCAAAAACAAAATCAGGCCAGGCTCTCTGTTTGAT
GAAGTCAGAAAGACGGCAAGGCTCAATCGGCGGCCCAGAAACCGCGAGAGCTCCAGTGAG
GAGGATTCTCCTGCACGAGAGAACAGCCCCTCCAGTAGCCGCAGCCGGTCACGGGCGAAA
TCGGATCCTAAATCCAGGCACAGAACAAGATCCCTCTCCTATAGTCACTCAAG

>NC054206_Scincella_reevesii
TCCCCAGTAAGTCTGAAGGTGAACTTCCAGGCTGCTGGCAAGAACTCCAATGCATCAAAT
AACTCTGAAGCAGGGCGTGCCAAGCAA---GAATTGGTTGGTGTGAAA
GGGAGCAGAAAAGAGAGGGGAAAACAAAGGCCCAGCCCCACTGCTGCAACTACTGTTGCA
GAAAGTGTGCCGAAAACGGACATGGCGGAAAATGCCCAGAGCAGCCTGGTGGATAAGAAA
TGGAAGCCACTGCAGGGTGTGGGGAATCTCCAGGTGGCTGCAGCAGCTGCAGCAGCTGCT
AAC-CCTGTCGAAGCCAAAAACATGGCATCATCGTCT---GACTCCAAACCA
CCAGGCTTAAGGATTGAGATCAAAAGCAAAAACAAAATTAGACCAGGCTCTCTGTTTGAT
GAAGTTAGAAAGACAGCCAGACTCAATCGGAGACCAAGAAACCGGGAGAGCTCCAGTGAG
GAGGATTCGCCTGCACGAGAGAACAGCCAGTCCAGGAGCCGCAGCCGGTCACGGAGCAAA
TCGGCTCCTAAACCCAGGCACAGAACAAGTTCCCCCTCCTACAGTCACTCAAG

>zeeshan_ZM146rag_rag1-F

TGACGCAGCCAGATGGCTAGCCACGATAAAGGGAATGGGGCCGATTCCCTGGACAAAGCCTCCGAGGAACAGGA
AGATGCAGTGGGCTTAAGGTCACAAGACCCCTTTCGAATAGACCGAGAGCGGAACACTGTTGTGGAGGCGAGAG
ATAAAGATGCCTTTCGTGTGAACCTAAGAGAGGATGAAGCTCACCAAGCAAAGCTGCAGCACCTCTGTCGAATCT
GTGGAGGCTCATTTAAAACTGACCCTTATAAGAGAAGCCACCCTGTGCATGGACCAGTGGATAATTTGATGCAGG
CCCTACTGAGAAAGAAGGAGAAAAGGGCAACATCTTGGCCAGAACTTCTTGCCAGGATTTTTAAGATTGATGTCA
GAGGAGACAATGATACCATCCATCCCACTAACTTTTGCCACAACTGCTGGAACGTGATTCAGAGGAAATTCAGCAA
TGTCCCTTGTGAAATGTATTTTCCAAGGAAAGGCGCTGTGGAGTGGCATCCCCATTCATCCAGTTGTGATGTTTGT
GGCACTTTATCCCGGGGGGTCAAGAGAAAGTGGCCAGCCCTGAATCCACAAGTGATCAAAAAAATCAGGATCCTC
GCCAGACCTGGTAGACAAACAAGGCAGGTAAAGAACCCCAAACAAGGGAATAGCAAAAGTCTCATGAAAAAGAT
TGCAAACTGCAACAAGATACATCTCAGTACAAAGAGCTTTGCAGTGGACTATCCTGCCGACTTTGTGAAATCTGTC
TCTTGCCAGATCTGTGAGCACATCCTGTCAGACCCGGTAGAAACAACATGCAAGCACTTATTCTGCAGAGTTTGCA
TCCTGAAATGCCTCAAAGTACTGGGAAGCTACTGCCCCAGTTGCCGCTATCCTTGCTTCCCTACTGATCTTGTGAGC
CCCGTAAAATCCTTCCTGAGCATCCTCAACAATTTGGCAGTGAGATGTCCTGTGAAAGAGTGTGATGAGGAAATCG
CTCTGGAAAAATACTGCCGTCATCTTTCTACCCACAAAGAAGCAAAGGACAAAGAA

>zeeshan_ZM145rag_rag1-F
TGACGCAGCCAGATGGCTAGCCACGATAAAGGGAATGGGGCCGATTCCCTGGACAAAGCCTCCGAGGAACAGGA
AGATGCAGTGGGCTTAAGGTCACAAGACCCCTTTCGAATATACCGAGAGCGGAACACTGTTGTGGAGGCGAGAG
ATAAAGATGCCTTTCGTGTGAACCTAAGAGAGGATGAAGCTCACCAAGCAAAGCTGCAGCACCTCTGTCGAATCT
GTGGAGGCTCATTTAAAACTGACCCTTATAAGAGAAGCCACCCTGTGCATGGACCAGTGGATAATTTGATGCAGG
CCCTACTGAGAAAGAAGGAGAAAAGGGCAACATCTTGGCCAGAACTTCTTGCCAGGATTTTTAAGATTGATGTCA
GAGGAGACAATGATACCATCCATCCCACTAACTTTTGCCACAACTGCTGGAACGTGATTCAGAGGAAATTCAGCAA
TGTCCCTTGTGAAATGTATTTTCCAAGGAAAGGCGCTGTGGAGTGGCATCCCCATTCATCCAGTTGTGATGTTTGT
GGCACTTTATCCCGGGGGGTCAAGAGAAAGTGGCCAGCCCTGAATCCACAAGTGAGCAAAAAAATCAGGATCCTC
GCCAGACCTGGTAGACAAACAAGGCAGGTAAAGAACCCCAAACAAGGGAACAGCAAAAGTCTCATGAAAAAGAT
TGCAAACTGCAACAAGATACATCTCAGTACAAAGAGCTTTGCAGTGGACTATCCTGCCGACTTTGTGAAATCTGTC
TCTTGCCAGATCTGTGAGCACATCCTGTCAGACCCGGTAGAAACAACATGCAAGCACTTATTCTGCAGAGTTTGCA
TCCTGAAATGCCTCAAAGTACTGGGAAGCTACTGCCCCAGTTGCCGCTATCCTTGCTTCCCTACTGATCTTGTGAGC
CCCGTAAAATCCTTCCTGAGCATCCTCAACAATTTGGCAGTGAGATGTCCTGTGAAAGAGTGTGATGAGGAAATCG
CTCTGGAAAAATACTGCCGTCATCTTTCTACCCACAAAGAAGCAAAGGACAAAGAA
